# Transient Lymphatic Remodeling Follows Sub-Ablative High-Frequency Irreversible Electroporation Therapy in a 4T1 Murine Model

**DOI:** 10.1007/s10439-024-03674-y

**Published:** 2025-02-25

**Authors:** Savieay Esparza, Edward Jacobs, Jennifer H. Hammel, Sharon K. Michelhaugh, Nastaran Alinezhadbalalami, Margaret Nagai-Singer, Khan Mohammad Imran, Rafael V. Davalos, Irving C. Allen, Scott S. Verbridge, Jennifer M. Munson

**Affiliations:** 1 Fralin Biomedical Research Institute at Virginia Tech-Carilion, Room 1210, 4 Riverside Circle, Roanoke, VA 24016 USA; 2 Department of Biomedical Engineering & Mechanics, Virginia Tech-Wake Forest School of Biomedical Engineering & Sciences, Blacksburg, VA USA; 3 Department of Biomedical Sciences and Pathobiology, Virginia-Maryland College of Veterinary Medicine, Blacksburg, VA USA; 4 Wallace H. Coulter Department of Biomedical Engineering, Georgia Tech & Emory University, Atlanta, GA USA

**Keywords:** High-frequency irreversible electroporation, 4T1, Lymphangiogenesis, Microvasculature, Breast cancer, Sub ablation, CCL21, Tumor-draining lymph node, VEGFC

## Abstract

High-frequency irreversible electroporation (H-FIRE) is a minimally invasive local ablation therapy known to activate the adaptive immune system and reprogram the tumor microenvironment. Its predecessor, irreversible electroporation (IRE), transiently increases microvascular density and immune cell infiltration within the surviving non-ablated and non-necrotic tumor region, also known as the viable tumor region. However, the impact of pulse electric field therapies on lymphatic vessels, crucial for T-cell fate and maturation, remains unclear. This study investigates how sub-ablative H-FIRE (SA-HFIRE) affects lymphatic and blood microvascular remodeling in the 4T1 mammary mouse model. We conducted a temporal and spatial analysis to evaluate vascular changes in the viable tumor, peritumoral fat pad, and tumor-draining lymph node post-treatment. Histological examination showed a transient increase in blood vessel density on Day 1 post-treatment, followed by a spike in lymphatic vessel density in the viable tumor region on Day 3 post-treatment, increased lymphatic vessel density in the peripheral fat pad, and minimal remodeling of the tumor-draining lymph node within 3 days following treatment. Gene expression analysis indicated elevated levels of CCL21 and CXCL2 on Day 1 post-treatment, while VEGFA and VEGFC did not appear to contribute to vascular remodeling. Likewise, CCL21 protein content in tumor-draining axillary lymph nodes correlated with gene expression data from the viable tumor region. These findings suggest a dynamic shift in lymphatic and blood microvascular structures post-SA-HFIRE, potentially enhancing the adaptive immune response through CCL21-mediated lymphatic homing and subsequent lymph node microvascular remodeling. Future work will assess the immune and transport function of the microvasculature to inform experiments aimed at the application of adjuvant therapies during scenarios of tumor partial ablation.

**Supplementary Information:**

The online version contains supplementary material available at 10.1007/s10439-024-03674-y.

## Introduction

The use of minimally invasive, focal ablative pulsed electric field (PEF) therapies is under investigation for multiple cancers [1–4]. One such therapy—high-frequency irreversible electroporation (H-FIRE)—utilizes high-voltage bipolar electric pulses on the microsecond scale to destabilize tumor cell membranes, leading to cell death non-thermally [5]. H-FIRE has seen use in treating breast [6], liver [7], and brain [8] malignancies in vivo and in a clinical study of prostate cancer [9]. Of interest is the specificity of the therapy to preserve proteinaceous structures such as nerve fibers [10], extracellular matrix, and mature vasculature [7], although tumor-associated vasculature may be more susceptible to electroporation than the vasculature of normal tissue [11].

Of importance, partial ablation within PEF therapy is a realistic concern for the clinical therapeutic application because it is an effect of electrochemotherapy application [12]. H-FIRE and other electroporation-based therapies rely on parameters such as electrode configuration, pulse width, and applied voltage to control ablation volumes [13]. Tissue heterogeneity, among other factors such as tissue conductance [14] and extracellular calcium [15], contributes to volumetric coverage of the applied electric fields, which can vary between patients and tumors, making consistent complete ablation unlikely. Despite the barriers to achieving a complete ablation, Ringel-Scaia et al. found even an incomplete ablation with H-FIRE can shift from an immune suppressive to pro-inflammatory tumor microenvironment, including enhanced antigen presentation and IL-17 signaling gene expression, following moderate response to H-FIRE ablation in a 4T1 mammary carcinoma mouse model [6]. Here, H-FIRE therapy elicited tumor volume reduction between 84 and 94% at 15 days post-treatment, suggesting enhanced adaptive immune system signaling despite incomplete tumor ablation. Interestingly, in a pancreatic cancer mouse model, IRE was shown to transiently enhance the microvessel density within the non-ablated viable region of the tumor following ablation [16, 17]. Zhao et al. showed an enhanced transport of dextran and immune cell infiltration within the tumor, which suggests altering the tumor microvasculature with PEF can benefit the anti-tumor immune response.

Vasculature dysfunction and expropriation in cancer are important for tumor progression and metastasis [18], therapeutic transport [19], and immune infiltration [20]. Tumor vasculature is leaky and in a constant state of angiogenesis induced by the tumor and microenvironment. Likewise, the lymphatic vasculature contributes to fluid homeostasis through drainage [21], metastasis [22, 23], and immune surveillance [24]. In the context of PEFs, treatment characterization has focused on the survival of large blood vessel architectures after ablation, cardiovascular tissue response, and even blood-brain barrier disruption [1, 25–28]. IRE and H-FIRE can also increase cytokines that promote an activated microvascular phenotype, such as IL-6, TNFα, and VEGFA [6, 29], while potentially eliciting anti-blood microvascular effects [30, 31].

Despite the breadth of information on blood vascular PEF effects, characterization of lymphatic-specific effects is lacking. A single study by Li et al. identified the effect of steep pulsed electric fields on lymphatic vasculature in a preclinical breast cancer rabbit model while maintaining the intention to ablate the vessels. They found decreased VEGFC immunohistochemistry (IHC) staining and intratumoral lymphatic destruction local to the ablation, as well as disrupted tumor drainage of methylene blue into surrounding lymphatics of the skin [32]. However, the timescale post-treatment was only reported within hours as compared to days. In vitro studies have shown that low direct current electric fields with an applied voltage-to-distance ratio of (1–3 V/cm) are capable of inducing lymphatic endothelial cell migration and activation of VEGF-3, suggesting enhanced lymphangiogenic potential [33, 34]. Taken together, the effects of either ablative or sub-ablative PEFs on the lymphatic system are unclear, and more work is needed to characterize the role of PEFs in lymphatic modulation. Thus, we sought to characterize the potential of sub-ablative H-FIRE (SA-HFIRE) therapy to induce transient lymphatic and blood microvasculature remodeling in vivo utilizing a mouse 4T1 mammary tumor model.

## Materials and Methods

### Cell Culture

Mouse mammary carcinoma cell line 4T1 was originally obtained from the American Type Culture Collection (ATCC). 4T1 cells were cultured in RPMI-1640 medium supplemented with 10% fetal bovine serum (FBS). All cell lines were grown in a sterile humidified incubator, kept at 5% CO_2_ and 37 °C. Cell lines were tested for mycoplasma prior to all experiments.

### Hydrogels and In Vitro HFIRE Application

Murine mammary carcinoma cells (4T1s) were seeded within 3D collagen tissue mimics to quantify their electrical lethal thresholds. Collagen scaffolds with final concentrations of 5 mg/mL were made from in-house lyophilized rat tail collagen [35]. A 10 mg/mL collagen stock solution was made by dissolving the freeze-dried collagen in 0.1% acetic acid. The collagen stock solution was mixed with 10X DMEM media (10% total volume, Sigma Aldrich) and 1N NaOH (~1% of total volume, Sigma Aldrich). NaOH was added dropwise and mixed until homogeneous to achieve ~ 7.4 pH, observed visually. 4T1s were then lifted from their flask and resuspended at 2.56 x 10^6^ cells/ml in supplemented media. The cell suspension (~39% total volume) was folded in to bring the collagen mixture to a final concentration of 5 mg/mL collagen with 1 x 10^6^ cells/ml. 220 µl of the collagen–cell mixture was then injected directly into the bottom of a treated 24-well plate (1.9 cm^2^, Corning), and the culture plates were then incubated for 25 min to allow for hydrogel polymerization. 440 µl of fresh media were added to cover each scaffold, and the culture plates were then placed again in the incubator for 24 h.

4T1-laden hydrogels were treated 24 h after seeding to allow the cells to engage with the extracellular matrix and mimic physiologically relevant morphologies. This allows for the cells to better follow ablation trends found in vivo and ex vivo [36, 37]. Applied electric fields were generated using a custom pulse generator (VoltMed Inc.), with applied voltage and current monitored as previously described (Supplemental Fig. 1a) [38]. Ambient temperature is shown to affect electroporation outcomes, so treatments were performed at 37 ºC within a miniature humidified incubator to increase the physiological relevance. The media were removed from the hydrogels, and bursts were delivered at 1Hz using two hollow needle electrodes (JG20-2.0T, Jensen Global) with a 3 mm center-to-center separation and a 500 V applied potential (voltage-to-distance ratio of 1666 V/cm). Directly after treatment, fresh supplemented RPMI-1640 was added to the wells, and the plate was again incubated for 24 h to allow for ablations to develop before imaging.

**Fig. 1:**
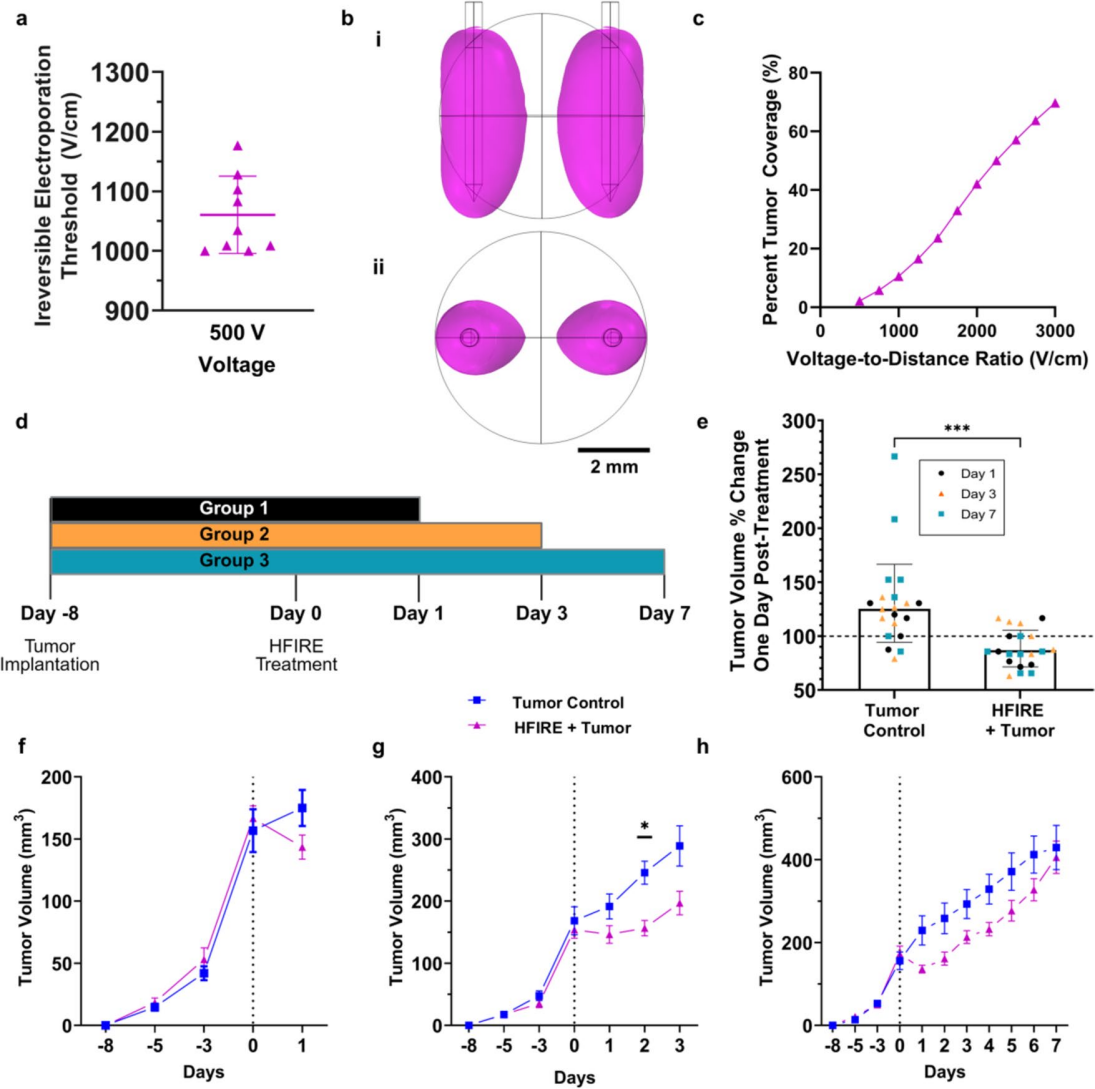
Tumor volume ablation is predictable with Sub-Ablative High-Frequency Irreversible Electroporation (SA-HFIRE). **a** Irreversible electroporation threshold of 4T1 cell-laden hydrogels. **b** The predicted ablation volumes for an applied voltage-to-distance ratio of 1500 V/cm applied electric field **(i)** front view with electrodes shown **(ii)** top view with electrodes as circles, from the 3D subcutaneous tumor model as designed in COMSOL. **c** The calculated percent tumor coverage in a 142 mm^3^ tumor for various applied electric fields. **d** In vivo experimental timeline and design. **e** Percent change in tumor volume 24 h after treatment with SA-HFIRE. **f** Tumor volume measurement over time for the Day 1 endpoint group with Day 0 treatment. **g** Tumor volume measurement over time for the Day 3 endpoint group with Day 0 treatment. **h** Tumor volume measurement over time Day 8 endpoint group with Day 0 treatment. All data shown as median with interquartile range as bars (*n* = 6–7 total per group). Dotted line represents treatment. All other data are shown as the Mean ± SEM. *p < 0.05, *** *p* < 0.001.

Cell viability was observed using a live/dead stain consisting of 2 μM of Calcein AM Green (Life Technologies) and 15 µM of propidium iodide (Life Technologies) in PBS. 440 µL of dye solution was used to completely cover the hydrogels and incubated for 30–45 min to allow complete diffusion through the hydrogel. The hydrogels were then washed twice with PBS to remove any remnant dye solution, and fluorescent tile-scan images were imaged using the Leica DMI8 microscope (Leica) with a 5x objective and 10x eyepiece. The live/dead images were analyzed within the Las X software. A custom-drawn region of interest was used to measure the areas of irreversible electroporation (Supplemental Fig. 1b,c). A 3D, time-dependent electric currents model (COMSOL™ Multiphysics 6.1) was created to replicate our in vitro set-up as done previously [13, 37] to calculate the electric field magnitude corresponding to the measured ablation area (Supplemental Fig. 1d).

### In Vivo Animal Study Design

All experiments were conducted under institutional IACUC approval and in accordance with the NIH Guide for the Care and Use of Laboratory Animals. 6–7 week-old female BALB/cJ (*n* = 7 animals in each group) were utilized. Mammary carcinoma 4T1 cells cultured as described above were washed, and resuspended in sterile PBS prior to injection at 1.2 x 10^6^ cells into the left abdominal mammary fat pad of anesthetized BALB/cJ mice. Clinical parameters and tumor growth were observed over 7 days, as previously reported [39].

### In Vivo H-FIRE Pulsing Parameters

H-FIRE therapy was delivered using the custom bipolar pulse generator EPULSUS 2.0 (Lisboa, Portugal) to apply 1500 V/cm voltage-to-distance ratio as 600 V applied across two monopolar 0.4 mm diameter acupuncture needle electrodes with 4 mm spacing and 4 mm electrode exposure. Therapy consisted of 200 bursts of bipolar pulses delivered at a frequency of 1 burst per second. Each burst was energized for a total time of 100 μs per burst with an individual burst of bipolar pulses with a 2 μs positive pulse, 5 μs inter-phase delay, 2 μs negative pulse, and a 5 μs inter-phase delay (2-5-2-5) scheme (waveforms are shown in Supplemental Fig. 1 g).

### In Vivo H-FIRE Treatment Design

On the eighth day of tumor growth at around 0.5–0.6 cm in diameter, half of the mice (*n* = 21) were anesthetized with isoflurane, and H-FIRE therapy was applied as a single insertion of the two-needle electrode per animal. After treatment animals are allowed to recover on air. Growth tracking continued until the endpoint day was reached at 1 day, 3 days, and 7 days post-H-FIRE therapy. On each day, 14 mice were euthanized (*n* = 7) from the H-FIRE treatment and (*n* = 7) from tumor-only age-matched control groups using CO_2_ inhalation. Mice were removed from the study if they were shown to not have received the correct pulsed electric field treatment application as determined at the time of treatment or if tumors grew into the peritoneal cavity.

### Tissue Harvesting and Processing

After euthanasia, tumor-bearing mammary fat pads containing inguinal lymph nodes were harvested, placed in plates to align the flat portion of the fat pad attached to the body cavity facing down, and post-fixed for 24 h in 4% formalin for paraffin embedding. Tumor-draining proper axillary lymph nodes were flash-frozen in liquid nitrogen for enzyme-linked immunosorbent assay (ELISA) analysis later.

### Immunohistochemistry, Immunofluorescent, and Picrosirius Red Staining

Formalin-fixed paraffin-embedded (FFPE) mammary fat pads were sectioned at 7 μm thickness. Serial sections on the same slide were deparaffinized and stained either with anti-podoplanin antibody (1 µg/ml, R&D Systems) and anti-CD31 (1 µg/ml, R&D Systems) for immunohistochemistry (IHC) or anti-LYVE−1 (1 μg/ml, Abcam) and anti-CD31 (1 μg/ml, R&D Systems) for immunofluorescence. For IHC, this was followed by ImmPRESS HRP anti-goat IgG peroxidase/SG peroxidase detection (Vector Labs) and nuclear counter-staining with hematoxylin (Vector Labs) to assess vessel density and area coverage. For immunofluorescence, anti-rabbit Alexafluor 488 and anti-goat Alexafluor 647 were used along with a DAPI counterstain at 1:5000. To assess collagen distribution, samples were stained with picrosirius red (PSR) kit (NovaUltra) according to supplier instructions. All samples were imaged using 20x objective on a VS200 slide scanner (Evident Scientific) for brightfield or fluorescent channels FITC, CY5, and DAPI, set to a constant exposure across samples. Polarized light was used for PSR samples with 20x objective and polarization filters set to 90° from each other.

### Semi-Automatic Vascular Analysis with ImageJ

Three regions of interest were selected per two distinct regions: viable tumor (V), represented by the tissue region outside of the necrotic and ablation area, and peritumoral fat pad (PT) for CD31^+^ and podoplanin^+^ stains, respectively (Supplemental Fig. 3b,1). To quantify area percent and vessel density, a custom FIJI (ImageJ) macro was utilized with the following workflow. The chromogenic images are transformed into RGB image space followed by color deconvolution set for H-DAB to isolate the HRP substrate brown signal in the image. Next, the IHC images were binarized using an intensity threshold using Max Entropy capable of isolating cross-sectional vessels with high specificity (Supplemental Fig. 3b;2i) generating a mask. The binary image is used to calculate the area percent of (CD31^+^ or podoplanin^+^) pixels from the image as well as converted into a green mask to be overlaid on the original image, enhancing visual identification for the user (Supplemental Fig. 3b;2ii). The overlaid image is used for counting cross-sectional vessels (CD31^+^ or podoplanin^+^) in a user-drawn region of interest (ROI). Vessel density is calculated as number of vessels in a region normalized to the tissue area of the region. For lymph node-specific stain quantification, the lymph node was similarly segmented including the sub-capsular space (Supplemental Fig. 3b;3,i-iii) followed by the same workflow. Area percentage was calculated from the binarized image normalized to the entire lymph node area.

### Lymph Node Semi-Automatic Vascular Analysis Using QuPath

QuPath version 0.4.4 [40] was utilized to analyze whole slide images of blood and lymphatic vascular stains: CD31, LYVE−1, DAPI; in the tumor-draining inguinal lymph node (Supplemental Fig. 3a). To quantify the lymphatic (CD31^+^LYVE−1^+^) vasculature and the blood (CD31^+^LYVE−1^−^) vasculature of the lymph nodes, a QuPath workflow was designed to segment, classify, and measure vascular stains in the inguinal lymph nodes. The workflow was as follows: inguinal lymph nodes were manually segmented from the whole mount tumor and mammary fat pad histological image. Manual removal of tissue artifacts was performed to decrease improper measurement of the stain intensity (Supplemental Fig. 3,a1). Next 0.25 mm^2^ ROIs from a sub-selection of 33% of the lymph nodes were separated into a Qupath pixel classifier training image spanning various locations within each respective lymph node (Supplemental Fig. 3,a2). The training image was used to train an individual pixel classifier to identify positive CD31 pixels with the following settings: an ANN_MLP artificial neural network consisting of 3 nodal layers with 3-5-2 nodes in each, 4 features (gaussian, hessian determinant, hessian max and min) at scales 2 and 4 from 0.65 µm resolution, equal parts of data from ignored pixels and CD31^+^ pixels, as determined by annotations of the CD31 stain channel, and boundary strategy set to one pixel classified as CD31 (Supplemental Fig. 3, a3). After classification, a manual check was performed to group vessel annotations improperly separated or separate vessel annotation with overlap between CD31 luminal vessels and LYVE−1 endothelium as shown as pink arrows in (Supplemental Fig. 3, a4). After this, an object classifier was used to classify vasculature as LYVE−1^+^ lymphatic vasculature or LYVE−1^−^ blood vasculature. The object classifier was trained on the same training images utilized by the pixel classifier. The object classifier consisted of a Random Trees (RTrees) model using equal distributed point annotations and seven features including ROI 2.0 µm CD31 pixel mean, median, and standard deviation of intensity, ROI 2.0 µm LYVE−1 pixel mean and standard deviation intensity, circularity, and solidity shape measurements. The resulting classified objects were then filtered based on 15 µm^2^ area to eliminate random background stains that made it through the process. A manual visual inspection was performed to remove vascular objects that had low mean intensity in CD31 and LYVE−1 channels based on background staining specific to that lymph node slice. The final classified vascular objects were measured and quantified for area, perimeter, and count within the lymph node annotation. Vascular complexity index (VCI) was defined previously as [41].

1 $$\frac{{\left( {\mathop \sum \nolimits_{i = 1,j = 1}^{n} P_{{\left( {i,j} \right)}} } \right)^{2} }}{{4\pi \mathop \sum \nolimits_{i = 1,j = 1}^{n} A_{{\left( {i,j} \right)}} }}$$

**Fig. 2:**
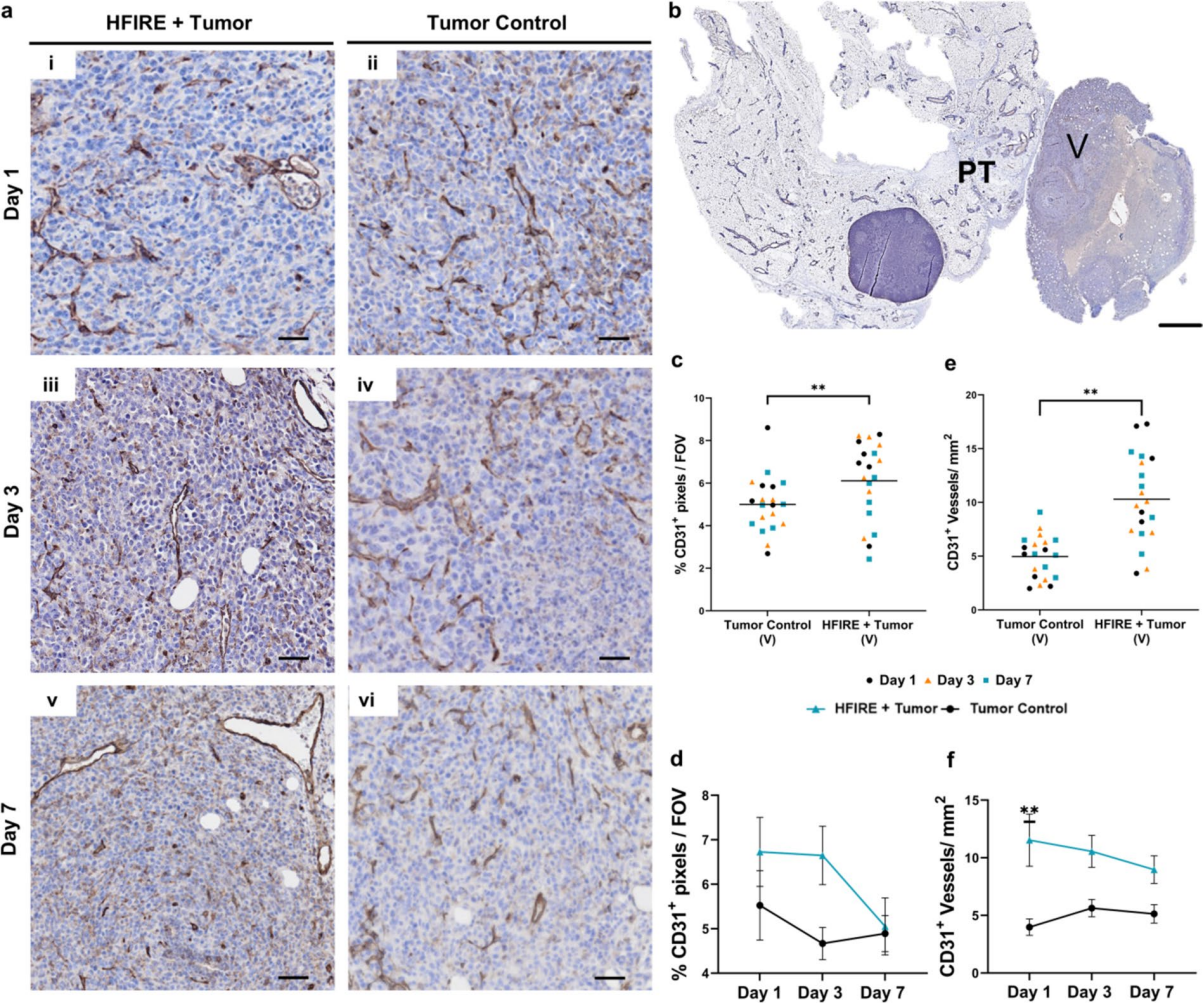
CD31^+^ microvascular transiently increases within the viable tumor region at 1 day after SA-HFIRE treatment. **a** Representative images of CD31 immunohistochemical staining of the viable tumor region separated by treatment with respective days **(i–ii)** Day 1, **(iii–iv)** Day 3, and **(v–vi)** Day 7. Scale bar represents 50 µm. **b** Representative whole mount image of CD31 staining of the viable tumor (V) and peritumoral fat pad (PT) regions in a SA-HFIRE treated Day 1 mouse. Scale bar represents 2 mm. All images show positive DAB signal (brown) counterstained with hematoxylin (blue). CD31^+^ area percentage of the viable tumor region, **c** combined and **d** separated by days. CD31^+^ vessel density of cross-sectional vessels, **e** combined and **f** separated by days. Scale bar represents 2 mm. *n* = 6–7 animals per group. *p < 0.05, **p < 0.01

**Fig. 3:**
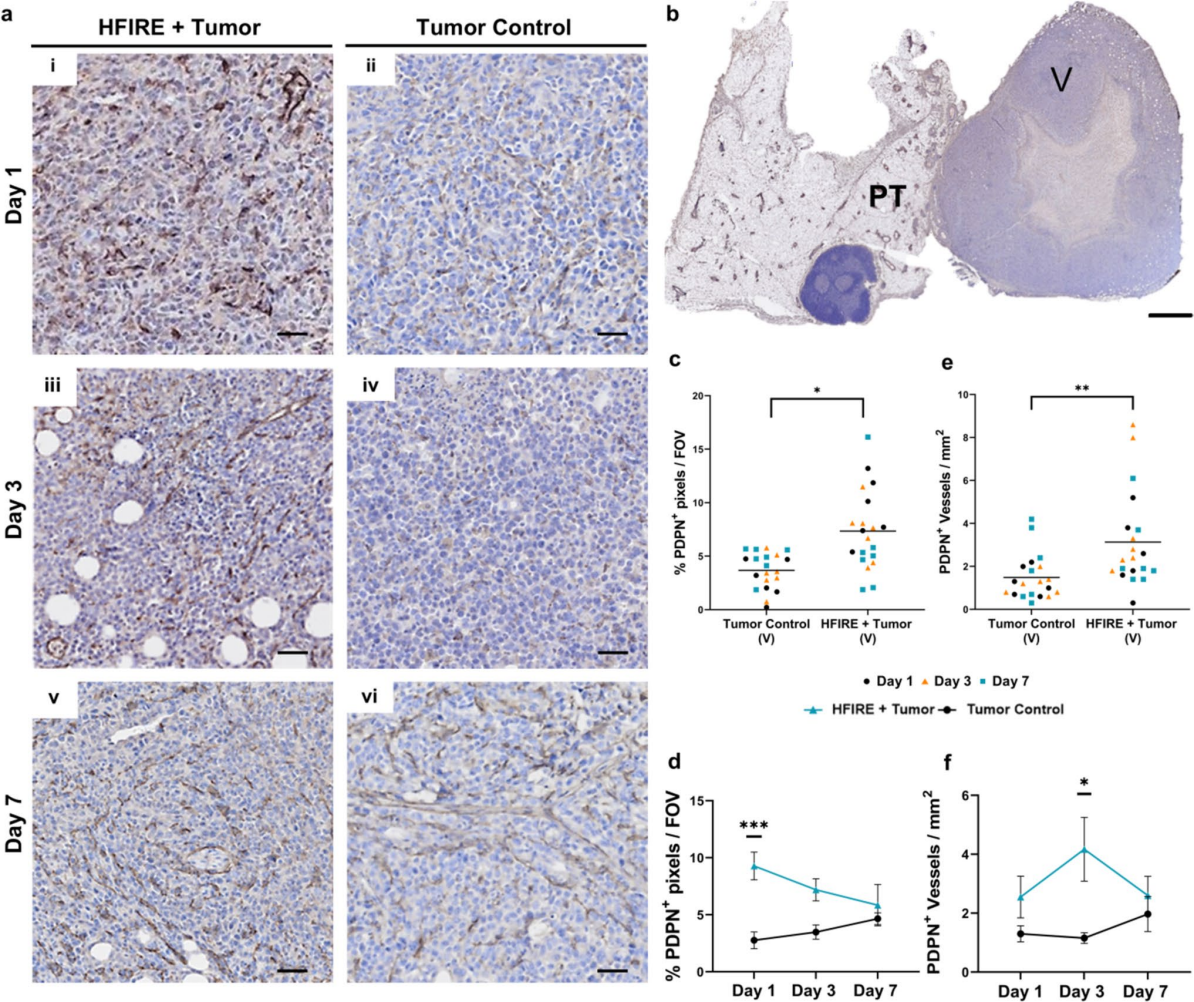
Podoplanin^+^ intratumoral microvasculature increases three days after SA-HFIRE treatment in the viable tumor region.** a** Representative images of podoplanin (PDPN) immunohistochemical staining of the viable tumor region by treatment with respective days **(i–ii)** Day 1, **(iii–iv)** Day 3, and **(v–vi)** Day 7. Scale bar represents 50 µm. **b** Representative whole mount image of the PDPN staining of the viable tumor (V) and peritumoral fat pad (PT) regions in a SA-HFIRE treated Day 3 mouse. Scale bar represents 2 mm. All images show positive DAB signal (brown) counterstained with hematoxylin (blue). PDPN^+^ area percentage of the viable tumor region **c** combined and **d** separated by days. PDPN^+^ vessel density of cross-sectional vessels, **e** combined and **f** separated by days. All data is shown as Mean ± SEM. *n* = 6–7 animals per group. *p < 0.05, **p < 0.01, ***p < 0.001.

**Fig. 4:**
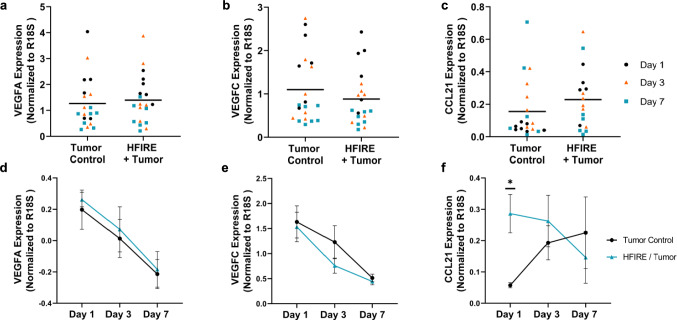
CCL21 gene expression in the tumor increases 1 day after SA-HFIRE treatment. Gene expression data in the tumor as a combination of all the days, for **a** VEGFA, **b** VEGFC, and **c** CCL21. Gene expression data in the tumor separated by day, for **d** VEGFA, **e** VEGFC, and **f** CCL21. All data are normalized to R18S measured using RT-qPCR from Tumor Control and HFIRE + Tumor, respectively. Data shown as Mean ± SEM. * *p* < 0.05. *n* = 6–7 animals per group

In short, VCI is calculated using both the total vessel annotation perimeter *P* and the total vessel annotation area *A* for each individual lymph node. The results were taken as the average of biological replicates in each group. For discretized computing, it is assumed a circle would result in 1–1.5711 for the ratio as the least complex shape.

### Quantitative Polymerase Chain Reaction (PCR) of FFPE Tumor and Mammary Fat Pads

Total RNA was harvested from FFPE samples of primary tumors and the surrounding mammary fat pad using Quick-DNA/RNA™ FFPE Kit following manufacturer's protocols (Zymo Research). RNA purity and concentration were measured using the Nanodrop Lite spectrophotometer system (Thermo Scientific). Prior to reverse transcription (RT), a pool of RNA from all samples with RNA concentrations above 300 µg/µl was used to create a standard curve consisting of 2000, 1000, 500, 250, 125, 61.25, 31.125 µg/µl RNA. RT was performed according to the manufacturer’s protocols using the QuantiTect Reverse Transcription Kit (Qiagen Cat: 205311), including a minus RT control reaction with no RT enzyme added. cDNA from each sample and the standard curve were used in the PCR amplification process using respective Taqman assays listed: R18s (Mm03928990_g1), CCl21a (Mm03646971_gH), VEGFA (Mm00437306_m1), VEGFC (Mm00437310_m1). Measurement of transcripts was performed with the Lightcyler96 system (Roche Diagnostics). Transcript abundance was calculated with a standard curve (*R*^2^ value > 0.9) replicated on each individual plate and was normalized to housekeeping R18S abundance.

### ELISA of Axillary Lymph Nodes

Total protein was isolated from flash-frozen proper axillary lymph nodes, dissected, and stored at the time of tissue harvesting. Lymph nodes were weighed prior to digestion and excess fat removed, followed by protein isolation according to manufacturer instructions. In short, lymph nodes were pulverized using BeadBug™, tissue homogenizers (Benchmark Scientific), before enzymatic digestion in the manufacture-provided buffers. After protein isolation, quantity was measured using a bicinchoninic acid (BCA) assay according to manufacturer instructions (Fisher Scientific, Pierce BCA kit). After quantification, CCL21 (SimpleStep, Abcam) and VEGFC (Abonva) ELISAs were performed according to the manufacturer’s instructions. Known concentrations of protein from mouse lymph nodes were loaded for each plate. Detection was performed on a CLARIOstar plate reader. A four-parameter logistic curve was used to determine the standard curve and concentrations of each sample. Samples were reported as picogram protein per milligram of lymph node tissue.

### Statistical Analysis

One-way or two-way analysis of variance (ANOVA) followed by Tukey’s multiple comparison test was used for statistical analysis of unmatched groups. Two group comparisons of normally distributed data as assessed by QQ plots were performed using unpaired* t* tests (with Welch’s correction if standard deviations were unequal). For lognormal distributed data, data were transformed by taking the log base 10 of the individual values. These transformed values were then assessed with previously mentioned statistical methods, and significance was marked with * on the original untransformed data presented in the respective figures. Statistical analyses were performed with GraphPad Prism version 10.0.0 for Windows (GraphPad Software). *p < 0.05* was considered statistically significant. Murine study numbers are noted in legends and by individual graphed data points. Graphs were generated using GraphPad Prism software and are shown with mean ± standard error of the mean, unless otherwise noted.

## Results

### Sub-Ablative H-FIRE (SA-HFIRE) Significantly Reduces Tumor Volume and Prevents the Regrowth of 4T1 Tumors for 7 Days

To determine an effective ablation volume for sub-ablative H-FIRE (SA-HFIRE) therapy, a multilayered COMSOL model of the tumor was developed using the irreversible electroporation threshold from 4T1 cell-laden hydrogel tissue mimics in vitro, as done previously (Supplemental Fig. 1a–d) [38]. The lethal electric field threshold was found to be 1077 V/cm (Fig. 1a) for 4T1s treated with a 2-5-2-5 H-FIRE waveform. The lethal threshold was used to estimate the ablation volume of the in vivo tumors at 0.65 cm in diameter corresponding to that measured on the day of treatment (Fig. 1b.i,ii; Supplemental Fig. 1d). Using the multilayered COMSOL model, tumor percent coverage was estimated for various voltage-to-distance ratios ranging from 500 V/cm to 3000 V/cm, which correspond to no ablation and above voltage-to-distance ratios previously used for therapeutic effects (Fig. 1c) [6]. The 1500 V/cm voltage-to-distance ratio selected showed an estimated tumor coverage of about 23.32% for the experimental tumor size.

To observe temporal effects of SA-HFIRE in vivo, an experimental design consisting of a treatment and control group with three post-treatment time points was chosen (Fig. 1d). This time frame was chosen to match prior work showing shifts in immune cell populations beneficial for tumor regression after H-FIRE treatment [6]. Twenty-one mice received SA-HFIRE treatment, with a single mouse receiving incorrect dosage and one mouse having incorrect tumor implantation, eliciting their removal from the study (Supplemental Fig. 2b, c). On Day 1 post-treatment, the percent change in tumor volume as compared to the day of treatment showed a significant average tumor volume increase of 135% for the tumor control group versus 80% in the SA-HFIRE group (Fig 1e), corresponding with the estimated 23.32% reduction from the computational model.

Similarly, the percent change in mouse weight followed a similar trending decrease among the treatment group (Supplemental Fig. 2a,e). The tumor growth was significantly reduced until the third day when both groups began showing similar tumor volumes through day 7 (Fig. 1f–h). Taken together, sub-ablative H-FIRE with a controlled ablation region, localized entirely within the tumor (Fig. 1b), allows for sizeable tumor reduction, although is not substantial enough to maintain tumor reduction across a 7-day period.

### SA-HFIRE Transiently Increases CD31^+^ Microvessel Density in the Viable Tumor Region 1 Day Post-Treatment

Prior work with IRE showed transient microvascular remodeling in a pancreatic murine mouse model within the surviving non-ablated and non-necrotic region of the tumor, referred to as the viable tumor region [16, 17]. To observe if SA-HFIRE could induce microvascular remodeling, we performed an immunohistochemical analysis of CD31 within the viable tumor region and the peritumoral fat pad (Supplemental Fig. 3b). Similar to IRE [16, 17], a transient increase in CD31^+^ area percent coverage and CD31^+^ cross-sectional vessel density was observed in the viable tumor region (Fig. 2c, e). CD31^+^ microvessels showed a qualitative increase in luminal areas with treatment (Fig. 2a). CD31^+^ vessel density, but not area coverage, significantly peaked 1 day post-treatment followed by a trending decrease similar to the control group by day 7 (Fig. 2b, d, f). In the peritumoral fat pad, CD31^+^ area percent coverage is significantly increased with SA-HFIRE treatment, although not vessel density (Supplemental Fig. 4a, b, d). CD31^+^ vessel density shows a trending peak at 3 days post-SA-HFIRE following the increase in CD31^+^ area coverage across all days as compared to the control group (Supplemental Fig. 4c, e). Although CD31^+^ vessels include both lymphatic and blood microvasculature, the data suggest transient vascularization and remodeling occurs locally in the viable tumor, but to a lesser extent in the peritumoral fat pad.

### SA-HFIRE Induced Intratumoral Lymphatic Remodeling Over 7 Days Following Blood Vascular Remodeling.

To resolve the lymphatic microvascular response from that of the CD31^+^ microvasculature in SA-HFIRE, we analyzed podoplanin (PDPN), a lymphatic-specific glycoprotein, within the viable tumor region and the peritumoral fat pad. Significantly increased PDPN^+^ area coverage and lymphatic cross-sectional vessel density were observed within the viable tumor after SA-HFIRE (Fig. 3c, e). PDPN^+^ area coverage peaked at 1 day post-SA-HFIRE (Fig. 3a, e), while PDPN vessel density peaked at 3 days post-treatment within the viable tumor region (Fig. 3d, f). Contrastingly, PDPN^+^ vessels in the viable tumor did not show enlarged lumens as compared to the CD31^+^ vessels (Fig. 3a, iii). SA-HFIRE treatment significantly increased PDPN^+^ vessel density in the peritumoral fat pad (Supplemental Fig. 4f, h), with PDPN^+^ vessel density peaking 3 days post-treatment (Supplemental Fig. 4g, i). The lymphatic vessels of the peritumoral fat pad showed no qualitative difference in luminal area compared with the CD31^+^ vessels (Supplemental Fig. 4d). Taken together, these data indicate that lymphatic vascular remodeling without lymphatic vessel enlargement occurs within the viable tumor and peritumoral fat pad after SA-HFIRE treatment.

### SA-HFIRE Induces Collagen Remodeling Within the Viable Tumor Region

Extracellular matrix remodeling in the tumor is a possible source of the microvascular remodeling observed after SA-HFIRE treatment and is associated with enhanced immune cell infiltration and transport following IRE [16, 17, 42]. To identify collagen network remodeling within the viable tumor, we utilized picrosirius red (PSR), a stain regularly used to identify collagen in tissues. A trending decrease in brightfield PSR area coverage was measured in the viable tumor region of SA-HFIRE-treated animals (Supplemental Fig. 5a, b) as compared to the control group. A trending decrease in brightfield PSR area occurred across the 7-day period with the largest difference from control animals occurring on day 7 post-treatment (Supplemental Fig. 5b, c). Using polarized light to identify red-yellow fibers from green fibers, thought to be representative of collagen I and collagen III respectively, we observed a trending shift in the ratio of red-yellow fibers to green fibers at day 7 post-treatment, although fewer total fibers were observed with this method as compared to the brightfield (Supplemental Fig. 5d). These data suggest that sustained collagen remodeling within the viable tumor may continue after peak vascular remodeling occurs with SA-HFIRE treatment. However, a more sensitive method of measuring collagen content should be used to confirm the extent of remodeling.

**Fig. 5:**
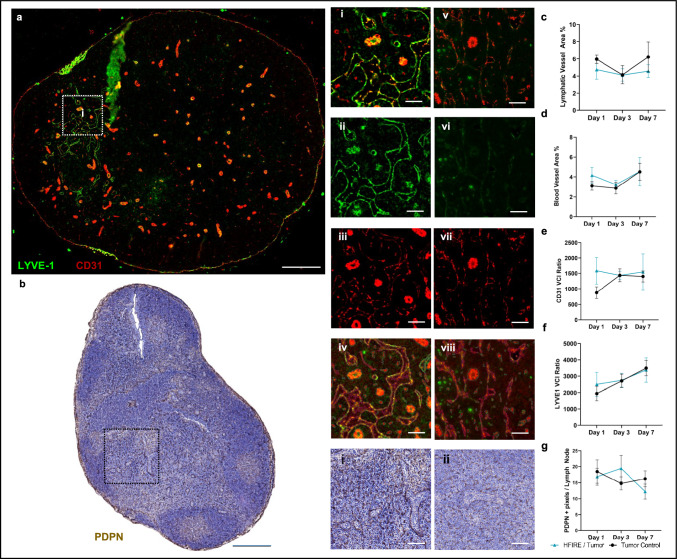
Lymphatic and blood vascular remodeling in the tumor-draining inguinal lymph node occurs 3 days post-SA-HFIRE treatment.** a** Representative immunofluorescence image of a Day 3 SA-HFIRE-treated inguinal lymph node. Scale bar represents 300 µm **(i)** Representative image of the vascular staining from (**a**) merged channels, **(ii)** LYVE−1 channel only, **(iii)** CD31 channel only, and **(iv)** QuPath classified vessels used for quantification. Scale bars represent 50 µm. **(v)** Representative image of the vascular staining of a similar location in a Day 3 Tumor Control inguinal lymph node merged, **(vi)** LYVE−1 channel only, **(vii)** CD31 channel only **(viii)** QuPath classified vessels used for quantification. Scale bars represent 50 µm. **b** Representative PDPN IHC image of a Day 3 SA-HFIRE-treated inguinal lymph node. Scale bar represents 300 µm. **(i)** Representative image of the PDPN stained fibroreticular cell network. **(ii)** Representative image PDPN IHC image of a similar location in a Day 3 Tumor Control inguinal lymph node. Scale bar represents 50 µm. **c** Lymphatic area percent coverage of the QuPath identified LYVE−1^+^ CD31^+^ vascular annotations normalized to total lymph node area after subtraction of artifacts. **d** Blood vessel area percent coverage of the QuPath identified CD31^+^ LYVE−1^−^ vascular annotations normalized to total lymph node area after subtraction of artifacts. **e** Blood vascular complexity ratio as a measure of total vessel network complexity. **f** Lymphatic vascular complexity ratio as a measure of total vessel network complexity. **g** PDPN area coverage quantification normalized to total lymph node area after subtraction of artifacts. All data are shown as Mean ± SEM. *n* = 6–7 animals per group.

### SA-HFIRE-Induced Vascular Remodeling is Not Due to Increased Angiogenic Gene Expression 1 Day Post-Treatment

To determine the contribution of prominent vascular growth factor, VEGFA, on the observed microvascular remodeling of the viable tumor post-SA-HFIRE, we measured gene expression of the tumor and fat pad using real-time quantitative PCR (RT-qPCR). A trending increase in VEGFA was found after SA-HFIRE treatment across all days within the tumor (Fig. 4a). However, a trending decrease in VEGFA gene expression occurred in parallel with the control group over the 7-day period (Fig. 4d). Similarly, in the surrounding fat pad, a trending increase in VEGFA gene expression is observed with a peak 1 day after treatment, although 15 animals, 7/20 in the treatment groups, and 8/20 in the control group did not have detectable VEGFA transcripts leading to reduced sampling. (Supplemental Fig. 7a, d). To interrogate other angiogenic factors that could be contributing to microvascular remodeling within the tumor at day 1, we applied an angiogenic real-time PCR-based gene expression array of 84 genes. Genes expression data showed trends of upregulation of 13 genes and down-regulation of 3 according to a 2-fold increase (Supplemental Fig. 6a). However, upon further inspection, only a significant 20-fold increase in *CXCL2* was observed in the treatment animals, while a range of 3-to-1-fold significant decrease was observed for *B2M, TIMP2, and FN1* (Supplemental Fig. 6b). Despite the overlap between genes contributing to angiogenesis and lymphangiogenesis, these data suggest that the vascular remodeling may not be associated with common angiogenic factors in SA-HFIRE at least at the tissue levels and timepoints tested.

**Fig. 6:**
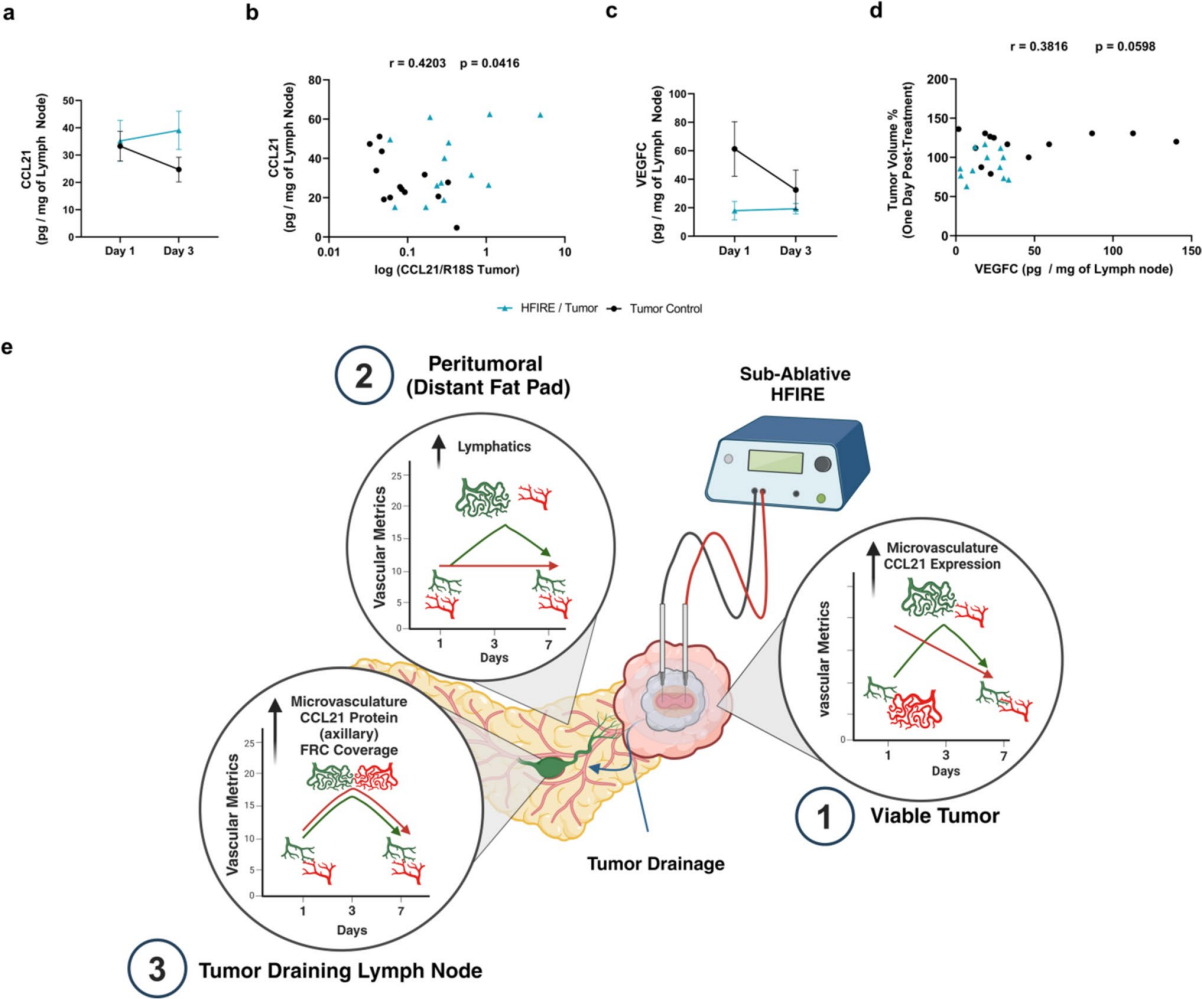
Dynamics of lymphatic and blood microvascular remodeling post-SA-HFIRE treatment.** a** Measured CCL21 protein normalized to axillary lymph node weight for 1- and 3-day post-SA-HFIRE treatment. **b** Correlation of CCL21 protein content of axillary lymph nodes to respective CCL21 gene expression in the tumor. **c** Measured VEGFC protein normalized to axillary lymph node weight for 1- and 3-day post-SA-HFIRE treatment. **d** Correlation of VEGFC protein content of axillary lymph nodes to the percent change in tumor volume 1 day post-SA-HFIRE treatment. Pearson Coefficient as r and* p* value are shown. **e** (1) Microvascular remodeling of the viable tumor region includes increased blood vasculature and CCL21 gene expression at one day post-SA-HFIRE, followed by intratumoral lymphatic remodeling three days post-SA-HFIRE. (2) Peritumoral/distant fat pad lymphatic remodeling occurs three days post-SA-HFIRE with little change to the blood vasculature. (3) Dynamics of the tumor-draining lymph node include lymphatic and blood microvasculature remodeling, increased CCL21 protein content of axillary lymph node, and increased fibroreticular cell coverage three days post-SA-HFIRE treatment.

### CCL21 Gene Expression Transiently Increases in the Tumor After SA-HFIRE Treatment

To probe lymphatic-specific growth factors, we quantified VEGFC expression, a prominent lymphatic growth factor, in the tumor and surrounding mammary fat pad using RT-qPCR. A trending decrease in VEGFC expression was observed within the treatment group (Fig. 4b), with the largest difference at day 3 post-treatment (Fig. 4e). Similarly, the mammary fat pad had a trending decrease in VEGFC gene expression across days until the seventh day post-treatment, although 17 animals, 6/20 in the treatment groups, and 11/20 in the control group, did not have detectable VEGFC transcripts leading to the reduced sampling (Supplemental Fig. 7b, e).

To probe functionality of initial lymphatic vasculature within the tumor, we measured the expression of CCL21, a lymphatic endothelium-specific cytokine that attracts antigen-presenting cells, T-lymphocytes, and conversely metastatic cancer cells through CCR7 activation [43, 44]. SA-HFIRE significantly increased CCL21 expression with a peak at 1 day post-treatment (Fig. 4c, f) and, subsequent trending decrease at days 3 and 7. Contrastingly, the mammary fat pad showed no significant change in CCL21 expression with treatment across days with a trending increase the seventh day post-treatment, although 15 animals, 6/20 in the treatment groups, and 9/20 in the control group, did not have detectable CCL21 transcripts leading to the reduced sampling (Supplemental Fig. 7c, f). These data suggest the dynamics of lymphatic remodeling within the tumor after SA-HFIRE are not directly related to VEGFC expression at the timepoints and tissue levels tested. Additionally, SA-HFIRE may contribute to lymphatic capillary immune functionality through CCL21 expression.

### SA-HFIRE Induces Transient Vascular Remodeling in the Tumor-Draining Inguinal Lymph Node

The tumor-draining lymph node (TDLN) is the primary site of antigen drainage and is associated with a shift from a pro-inflammatory to an immunosuppressive environment during cancer progression [45]. This shift has been associated with corresponding changes in lymph node vascular and stromal remodeling. Thus, we sought to identify if lymphatic, blood vascular, and stromal remodeling was present in the inguinal TDLN after SA-HFIRE. We developed a method for analyzing whole slide images in QuPath to identify LYVE−1^+^ CD31^+^ lymphatic vasculature from LYVE−1^-^ CD31^+^ blood vasculature (Fig. 5a, iv, vii, Supplemental Fig. 3a), as podoplanin shows co-expression by stromal fibroreticular cells and lymphatic endothelial cells within the lymph node. TDLNs treated with SA-HFIRE showed a trending decrease in total lymphatic vessel area (Fig. 5c). Notably, a qualitative increase in LYVE−1 stain with a punctate distribution was observed across the lymphatic endothelium in the TDLNs of treated animals as compared to the control animals (Fig. 5ai, ii, v, vi) although not directly reflected in the QuPath quantification. However, no qualitative difference in CD31 expression appeared between groups (Fig. 5a, iii, vii). Contrastingly, total blood vessel area showed a trending increase with treatment and with a peak 1 day post-treatment (Fig. 5d).

Qualitatively, SA-HFIRE TDLNs appeared to have larger vascular perimeters, so we sought to characterize the geometric parameters of the vascular networks. We utilized the vascular complexity index (VCI), a measurement of the vascular network’s perimeter to area, previously used to identify differences in the complexity of retinal vascular beds [41]. The VCI for the lymphatic and blood vasculature, although not statistically significant, showed a transient increase 1 day after SA-HFIRE treatment (Fig. 5e, f), with the lymphatic vascular maintaining over the 7-day period and the blood vasculature overall increased between groups. These measurements reflect the overall morphological changes in the vascular networks as lumens were larger in the high endothelial venules and the lymphatic endothelium of the medullar region 1 day after treatment. Together, these data suggest that SA-HFIRE treatment can alter the vascular phenotype in the TDLN contributing to shifting vessel morphology.

### Tumor-Draining Inguinal Lymph Node Stromal Normalization Occurs Post-SA-HFIRE Treatment

The stromal cells of the TDLN, specifically the fibroreticular cells (FRC), contribute to the localization of immune cells and the expansion of the reticular collagen network during an immune response [46, 47]. Thus, we sought to determine if FRC coverage and collagen remodeling occur in parallel to vascular remodeling. Using IHC staining for PDPN, here a marker expressed both on FRCs and the lymphatic endothelium in the LN, we observed a trending increase in PDPN^+^ area coverage across all days (Fig. 5b). This increase peaked at day 3, contrasting the dynamics of the vasculature, which may be attributed to FRC specific coverage. Qualitatively the PDPN^+^ FRCs morphology appeared more interconnected to adjacent FRCs as compared to that of the control animals with less distance in between adjacent FRCs (Fig. 5b,i,ii).

Utilizing PSR stain to identify collagen network remodeling within the TDLN, a trending decrease in brightfield PSR area coverage was measured in the SA-HFIRE-treated TDLNs compared to the controls (Supplemental Fig. 8b). The brightfield PSR area remained constant between days with the largest difference from control animals occurring on day 1 post-treatment (Supplemental Fig. 8c). Using polarized light, we observed a trending shift in the ratio of red-yellow fibers to green fibers, although fewer fibers were observed over the entire TDLN with this method (Supplemental Fig. 8e). Despite these changes in the collagen structure of the TDLN, no distinguishable difference was observed in TDLN tissue cross-sectional areas used in the quantification of PSR and IHC-stained samples across time and treatment (Supplemental Fig. 9a, b). Thus, FRC coverage appears to not be altered by SA-HFIRE, while the overall collagen network is reduced suggesting a shift in the lymph node stromal microenvironment, toward a phenotype supportive of an immune response [48].

### SA-HFIRE Increases CCL21 While Decreasing VEGFC Downstream of the Tumor in the Axillary Tumor-Draining Lymph Nodes

The proper axillary lymph nodes (PALNs) contribute to downstream drainage of the tumor and the inguinal lymph node providing a useful representation of protein content draining from both sites. CCL21 and VEGFC were measured using ELISA to assess the protein content of downstream PALNs at day 1 and 3, based on previous vascular remodeling in the tumor and TDLNs. A trending increase of CCL21 was measured at day three as compared to day 1 in SA-HFIRE-treated mice (Fig. 6a). VEGFC was reduced in PALNs of SA-HFIRE mice with little difference between day 1 and day 3 (Fig. 6c), reflective of decreased VEGFC gene expression within the tumor. The PALNs did not show significant changes in weight over both days, suggesting little activation and expansion as seen in immune responses or fluid accumulation. The CCL21 protein content significantly correlates with the CCL21 gene expression measured in the tumor (Fig. 6b). Similarly, the VEGFC protein content, although not significant at the chosen alpha of 0.05, clusters with the percentage change in tumor volume using correlation analysis (Fig. 6d). Taken together this suggests CCL21 and VEGFC protein content in tumor-draining PALNs shifts in a predictable manner with mRNA expression of the tumor and with the extent of ablation of the tumor after SA-HFIRE.

## Discussion

Here, we have utilized SA-HFIRE to interrogate the impact that PEFs have on lymphatic vascular remodeling through intentionally limiting tumor coverage of the ablation without inducing cell death of these structures. SA-HFIRE induced spatiotemporal dynamic vascular remodeling locally in the tumor, distally in the mammary fat pad, and to a lesser extent in the tumor-draining lymph nodes. We propose that this in part occurs via complex intercellular interactions, likely not due to common growth factors VEGFA or VEGFC, at least within the timeframe and tissue levels tested. Contrastingly, CCL21 and VEGFC gene expression, as well as CCL21 and VEGFC protein drainage to lymph nodes are important biomarkers of the SA-HFIRE-induced vascular remodeling and extent of ablation within the tumor (Fig. 6e).

In summary, the vascular remodeling dynamics observed following SA-HFIRE within the viable tumor mirrors similar remodeling as seen following IRE treatment [16, 17]. The transient increase in CD31^+^ vessel density is characteristic. The increased lumens of the blood vasculature could be evidence of decreased compression, possibly related to the collagen remodeling observed through reduced PSR stain over the 7-day period. Zhao et al. found down-regulation of lysyl oxidase-like protein, a collagen cross-linker, occurred after IRE in a pancreatic murine model which contributed to enhanced transport of dextran and immune cell infiltration into the tumor at a timepoint corresponding with maximum microvascular density [16]. Conversely, in the context of breast cancer, lysyl oxidase-like protein 2 contributes to lymphangiogenesis and lymph node metastasis [49]. Additionally, Zhao et al. found that changes in fibroblast activation protein alpha (FAPa^+^) fibroblast content within the viable tumor were associated with remodeling after IRE [16]. It is possible H-FIRE may cause a similar shift, although we did not stain for fibroblasts directly. Since PDPN co-stains fibroblasts within the tumor [50], it is possible that the transient increase in PDPN^+^ area coverage we saw in the tumor is reflective of this change in the population. For this reason, the PDPN^+^ vessel density may be a better indicator of the lymphatic remodeling within the tumor microenvironment, and an additional fibroblast-specific marker, such as α-smooth muscle actin, could be used to distinguish between the populations.

Despite the SA-HFIRE-induced changes in lymphatic vessel density, no observable change in PDPN^+^ vessel luminal area or perimeter was observed, which are indicative of lymphatic vessel enlargement as seen in inflammatory conditions to accommodate increased interstitial fluid drainage [51]. To better identify the lymphatic vessels, and thus the lymphatic remodeling within the tumor and surrounding periphery, alternative established markers such as LYVE−1 in conjunction with CD31 may be used to identify the vessels as was done within the lymph node vessel analysis. To understand if endothelial cell proliferation is a main factor in lymphatic remodeling, future work should consider the application of flow cytometry to provide better cellular specificity despite sacrificing the spatial information. Additionally, this stain combination with F4/80 can be leveraged to identify LYVE−1 positive macrophages which are associated with inflammatory lymphatic remodeling [52]. Although during inflammatory lymphatic remodeling the production of VEGFA and VEGFC is expected by activated macrophages, there was no observable increase of either within the treated tumor. Despite this, future work should consider the involvement of macrophages in the H-FIRE-induced microvascular remodeling, as prior work with H-FIRE has shown temporal reduction in tumor-associated macrophage populations following treatment [6].

The dynamics of lymphatic remodeling within the viable tumor after H-FIRE is a novel finding. Previous work by Li et al. used steep field pulsed electric fields with a pulse duration similar to the one studied here (2.6 µs vs. 2 µs) [32]. However, the voltage-to-distance ratio of the electric field applied was 400 V/cm at 100 Hz for 30 min, while both IRE and H-FIRE apply significantly less energy to induce cell death with typically 70–200 pulses applied at 1 Hz. Li et al. indicated that complete destruction of the lymphatics occurred within and in close proximity to the ablation, although due to the applied energy, it is likely that these intratumoral lymphatics were destroyed through thermal mechanisms that are fundamentally distinct from IRE. Following this, Li et al. found methylene blue dye injected into the surrounding skin of the tumor did not localize to peritumoral lymphatic vessels in the treated group, as compared to the control, suggesting the acute dysfunction of the lymphatics after steep field pulsed electric field application.

Of note, our temporal assessment differs from Li et al, as our study probed an extended 7-day period of time after ablation as compared to hours post-ablation in their study, limiting our interpretation of our results in the context of their work. Further, we saw an increase in peritumoral fat pad lymphatic density three days after treatment. It is possible the decreased physical compression as observed through the trending decrease in PSR staining of the viable tumor may contribute to intratumoral lymphatic vessel drainage of the tumor. This is in conjunction with the increase in peritumoral lymphatic vasculature, which is expected to contribute to an increase in functional drainage of interstitial fluid flow and immune suppression, as prior work has found [53]. When observing the most prominent lymphangiogenic growth factor, VEGFC, our results indicate a reduction in VEGFC gene expression similar to the reduction in VEGFC IHC stains Li et al. observed. Lymphatic survival and remodeling from H-FIRE treatment, although not likely dependent on VEGFC at the time scale and tissue level observed in this study, could contribute to the understanding of previous work through the measurement of tumor drainage following H-FIRE application.

Additionally, Ringel-Scaia et al. previously found enhanced gene expression of antigen presentation, IL-17, and pro-inflammatory signaling in moderately responsive 4T1 murine models, suggesting that adaptive immune system engagement is dependent on the extent of ablation following H-FIRE [6]. Since lymphatic vasculature contributes to the initialization and maintenance of an adaptive immune response, characterizing the inflammatory lymphatic vessel activation following H-FIRE could contribute to understanding the mechanisms of action in the tumor microenvironment. One such marker, CCL21, is released from initial lymphatic capillaries and can contribute to immune activation by attracting antigen-presenting cells and T-lymphocytes to localize to the lymph node [54, 55]. It is possible the increased CCL21 gene expression in the tumor and CCL21 protein content in the axillary lymph node are reflective of enhanced lymphatic capillary immune functionality within one to three days after SA-HFIRE, respectively. However, CCL21 expression from lymphatic endothelial cells must be defined to distinguish lymphatic-specific contributions from those of macrophages and the tumor cells themselves.

Likewise, a reduction in collagen and fibroblastic reticular cells in the TDLN is associated with contributions from other stromal cell components of the TDLN that can aid in an adaptive immune response [48, 56, 57]. Specifically, the lymphatic and blood vasculature of the TDLN play a dualistic role in contributing to immune responses and supporting immune suppression [58, 59]. Although no significant quantifiable differences were observed in the TDLN using the IHC image processing methods in QuPath, qualitative phenotypic differences were observed between the lymphatic and blood vasculature including larger lumens of HEVs and increased expression of LYVE−1 on the lymphatic medulla. This is possibly reflective of the nature of the more conservative image processing pipeline utilized, which segregated individual LYVE−1 signals taken only from CD31 positive vessels within a thin section of tissue. More elaborate methods of quantification using 3D tissue-cleared lymph nodes imaged with a light sheet microscope and segmented for these respective markers may overcome these limitations by maintaining physiological structure better.

Interestingly, these phenotypic changes are associated with T-lymphocyte extravasation from the HEVs into the lymph node parenchyma during an immune response and inflammation [47, 60]. The measured VCI of the vasculature within the TDLN at 1 day after SA-HFIRE suggests remodeling may only occur through the enlargement of vessels as the VCI is sensitive to changes in perimeter and less sensitive to area, which corresponds with the larger lumens observed. Although novel in this context, in retinal vasculature quantification, VCI is useful in describing the complex nature of the vasculature more reliably than other metrics of vascular quantification [41]. As the vasculature in the lymph node is highly irregular and difficult to measure using methods of vessel density and other image processing methods, to truly quantify changes in populations of stromal cells of the lymph node, flow cytometry, as done previously to identify lymphatic and blood endothelial cell populations [59], could detect the population shifts associated with SA-HFIRE, while sacrificing the morphological and spatial information. Despite these limitations, the TDLN vascular remodeling highlights the importance of identifying the lymphatic vascular contribution to the immune response after H-FIRE treatment.

Therapeutically, we show the application of sub-ablative H-FIRE at a voltage-to-distance ratio of 1500 V/cm is sufficient to induce significant tumor ablation while sparing a portion of viable tumor that undergoes microvascular remodeling. Additionally, vascular remodeling occurs in distal sites such as the peritumoral fat pad and TDLN to a lesser extent over a 7-day period. Interestingly, this microvascular remodeling is likely not associated with VEGFA or VEGFC. Likewise, lymphatic vascular remodeling shows the potential for an inflammatory phenotypic shift through CCL21 gene expression in the tumor, and LYVE−1 immunofluorescent staining within the TDLN. When considering the dynamics of microvascular remodeling, SA-HFIRE could benefit from the application of increased ablation coverage or the investigation of co-therapies as recent work has shown to enhance cell death and initiate the immune shift with combinatorial therapy [8]. Future work must assess the vascular immune function and characterize the transport within the tumor which could help to inform optimal timing for the application of adjuvant therapy, when considering sub-ablative H-FIRE. Overall, we have shown the transient remodeling of lymphatic endothelium, independent of VEGFC and the upregulation of a lymphatic endothelium-specific T-lymphocyte attracting cytokine, CCL21, one day after SA-HFIRE, showing the importance of considering the lymphatic system in association with H-FIRE.

## Supplementary Information

Below is the link to the electronic supplementary material.

Supplementary file1 (DOCX 34286 KB)

Supplementary file2 (DOCX 95 KB)
