# Supplementary material for: Transient Lymphatic Remodeling Follows Sub-Ablative High-Frequency Irreversible Electroporation Therapy in a 4T1 Murine Model
Source: Ann Biomed Eng. 2025 Feb 25;53(5):1148–64. doi: 10.1007/s10439-024-03674-y (PMC12006248; doi:10.1007/s10439-024-03674-y)
Supplement: Supplementary file 1 — Supplementary file1 (DOCX 34286 KB) [file 10439_2024_3674_MOESM1_ESM.docx]

# Supplemental Figures and Tables


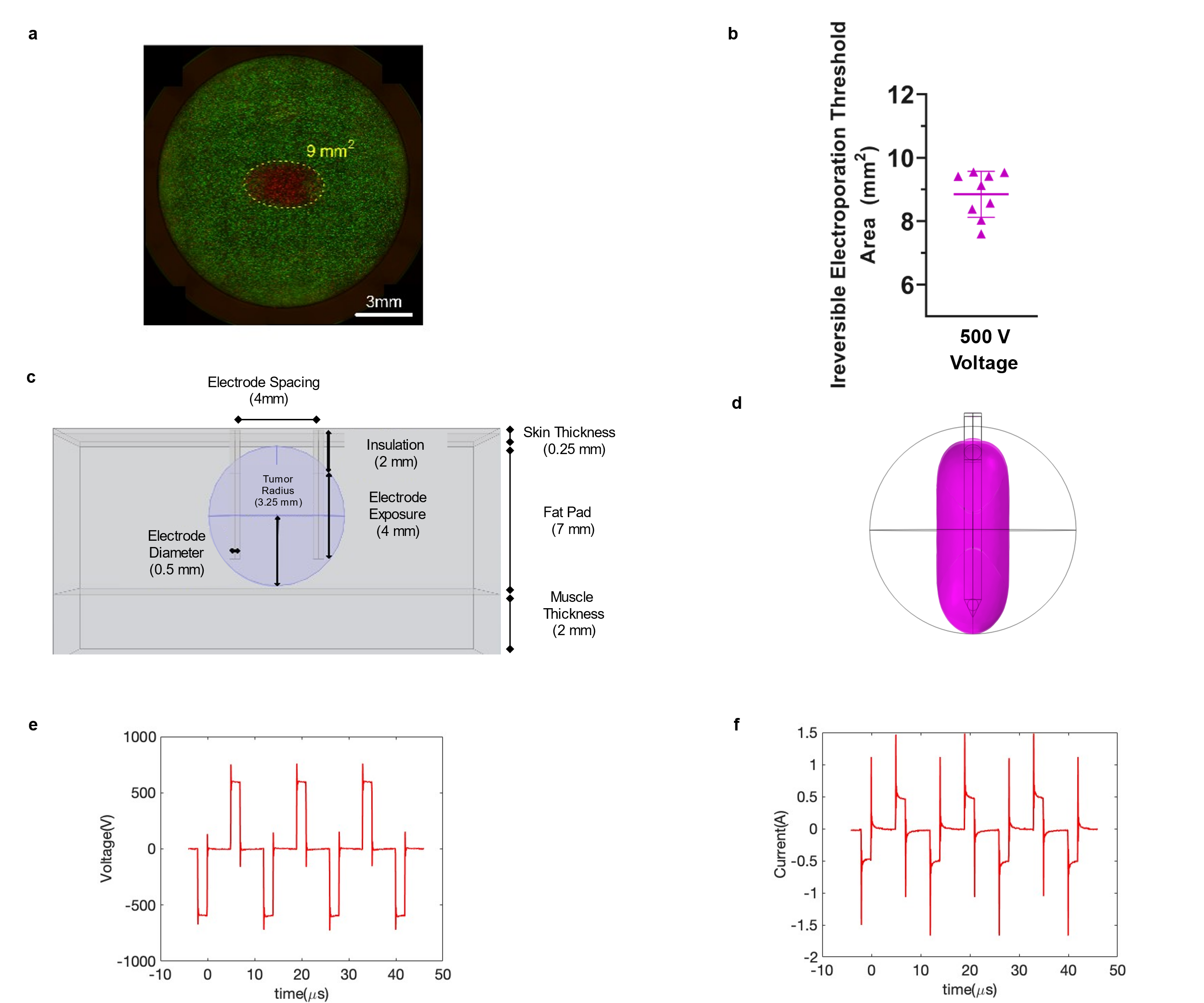


**Supplemental Figure 1. Waveform-dependent treatment predictions were established using *in vitro* 4T1 lethal thresholds in hydrogel tissue mimics.** 4T1-laden hydrogels were treated with a 500V applied potential, delivered using a custom high voltage pulse generator as done previously by Jacobs et al. **(a)** Ablations were developed over 24 hours and imaged using a live (Calcein AM, green) and dead (Propidium Iodide, red) stain in PBS. Ablation areas were measured with clear borders delineating the ablation, and lethal thresholds were calculated for each measured ablation area. **(b)** Corresponding Irreversible electroporation threshold represented by area of propidium iodide in the cell laden hydrogel tissue mimics. **(c)** A subcutaneous tumor was modeled with a multilayered system, consisting of muscle, fat pad, and skin. The electrode exposure and center-to-center space were both 4 mm, to match the experimental procedure, and the tumor was modeled based on tumor subcutaneous measurements at the time of treatment. **(d)** Side view of a single electrode showing the predicted ablation volume as determined by the COMSOL model. Example section of the oscilloscope output during one treatment regime showing **(e)** the current as applied during the pulsing scheme and **(f)** the Voltage applied across a subsection of the pulse regime.


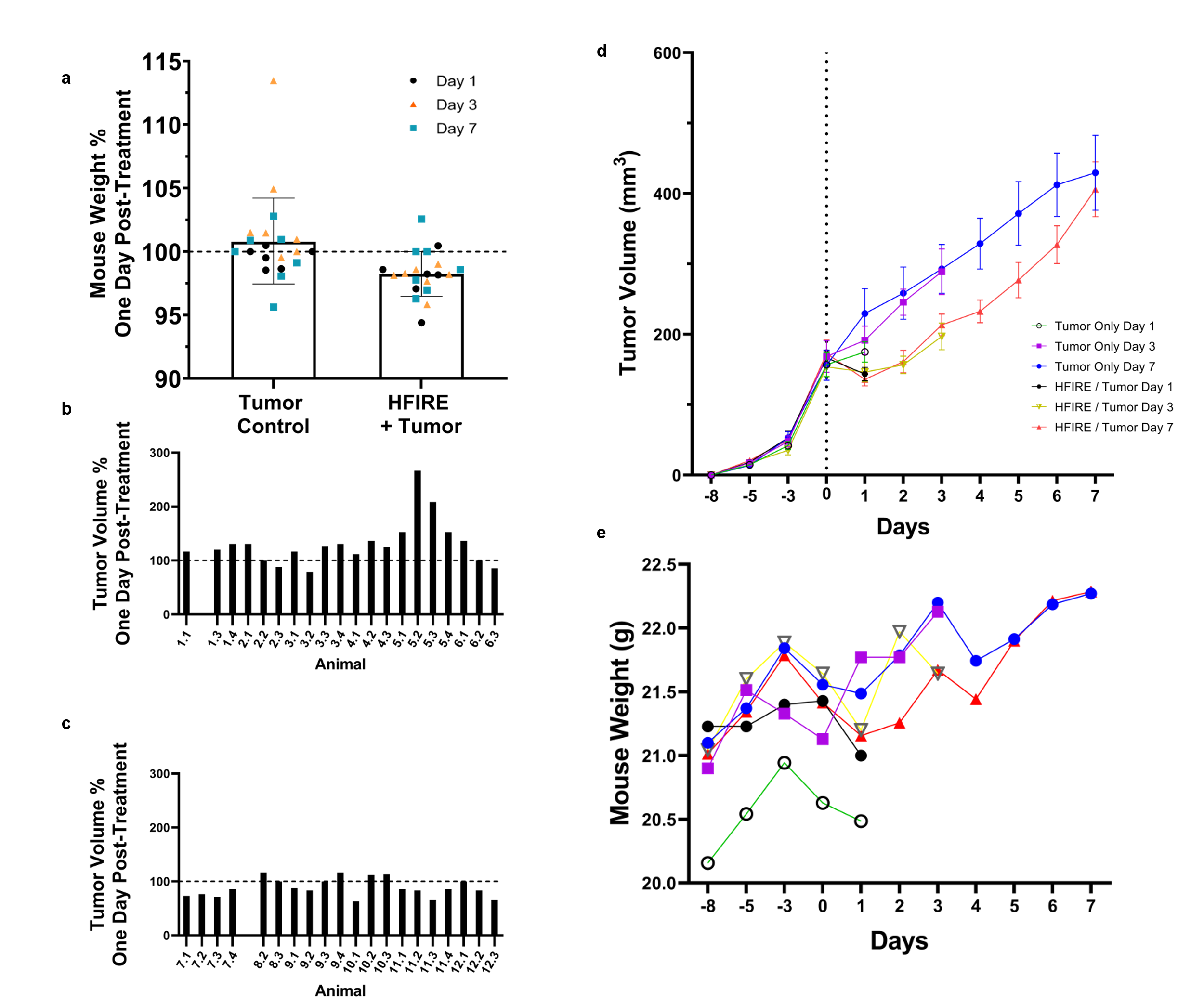


**Supplemental Figure 2. Mouse weight changes with SA-H-FIRE treatment. (a)** Percent change in the mouse weight shows the change in HFIRE treated mice and the immediate change within 24 hours post SA-H-FIRE treatment. **(b)** Percentage change of tumor volume shown for each individual animal used in the control treatment groups. All data shown as % change in tumor volume from day prior to treatment. **(c)** Percentage change of tumor volume shown for each individual animal used in the HFIRE treatment groups. **(d)** All animal tumor volumes plotted for respective groups. The dotted line is the day of treatment on day 0. **(e**) Mean Mouse weights for each group. Data shown as mean ± SEM.


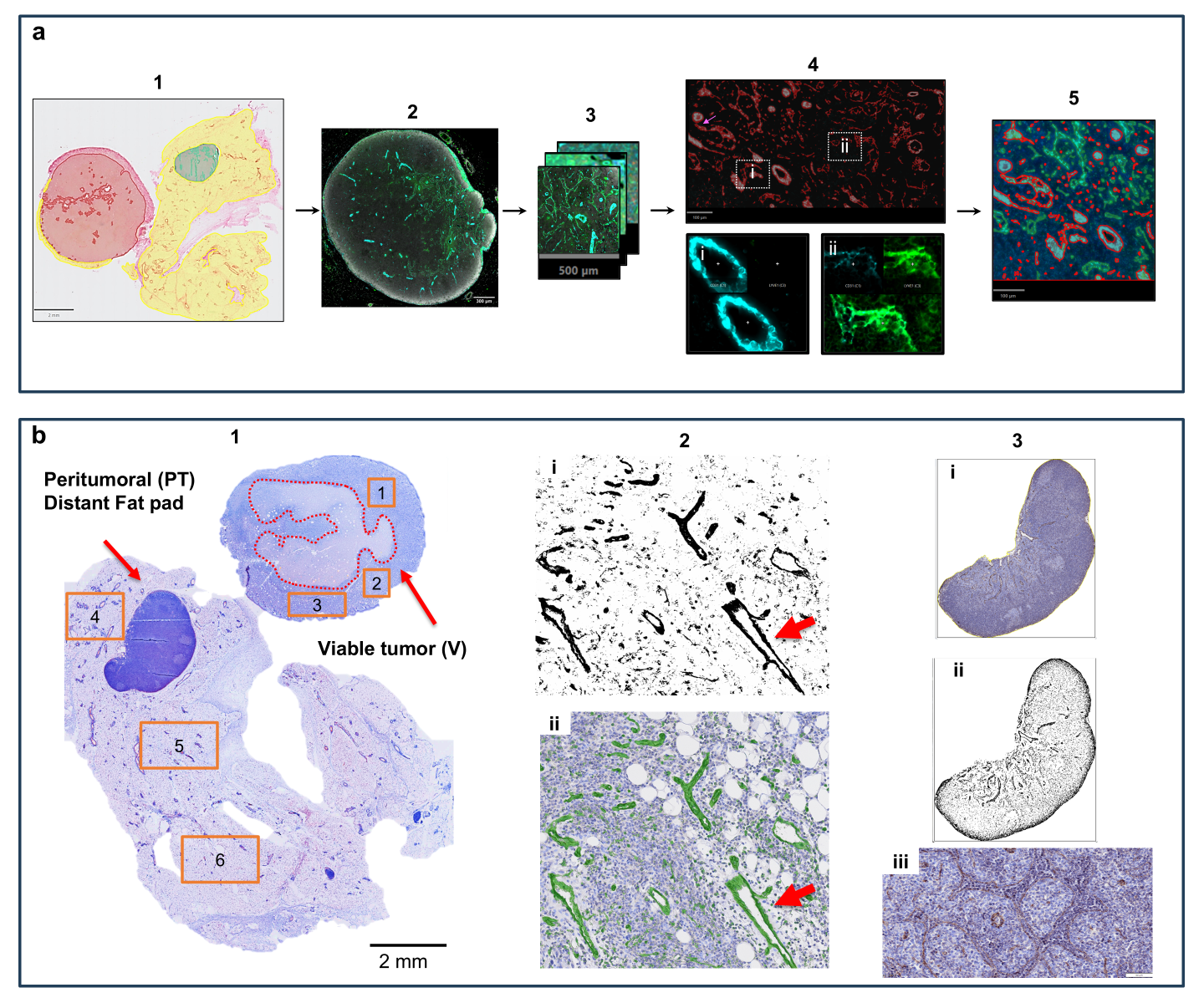


**Supplemental Figure 3. Pipeline used for vascular quantification in QuPath and FIJI.** **(a)** (1) Segmentation of a mammary fat pad section with the tumor (red) excluding skin tissue, fat pad (yellow), and inguinal lymph node (green). (2) Image of fluorescent stained lymph node with DAPI (white), LYVE-1 (green), and CD31 (cyan). (3) Example training images 500 µm x 500 µm. (4) Image showing the vasculature within the lymph node with QuPath overlay after CD31 pixel classification for different vasculature **(i)** CD31^+^ blood vessel (cyan) and **(ii)** LYVE-1^+^ lymphatic vessel (green). (5) Classification of vessels in QuPath after object classification of CD31^+^ annotations showing lymphatic vessels (green) and blood vessels (red). **(b)** Processing pipeline for vessel quantification in FIJI. (1) ROIs were selected from the viable tumor region (region of tumor excluding the ablation and necrotic regions), denoted (V) and the peritumoral / distant fat pad region denoted (PT) as shown by the arrows, from immunohistochemistry (IHC) stained serial sections stained with either podoplanin or CD31. (2) Representative image ROIs showing the I) mask and II) overlay of the pseudo-colored green mask on top of the original IHC image prior to vessel density quantification. The arrows point to an example of a cross-sectional vessel counted in the vessel density quantification. (3) Example images of (i) an inguinal lymph node section used in PDPN quantification after segmentation, (ii) mask image for PDPN stain, and (iii) PDPN stained vessel network and fibroreticular cells.


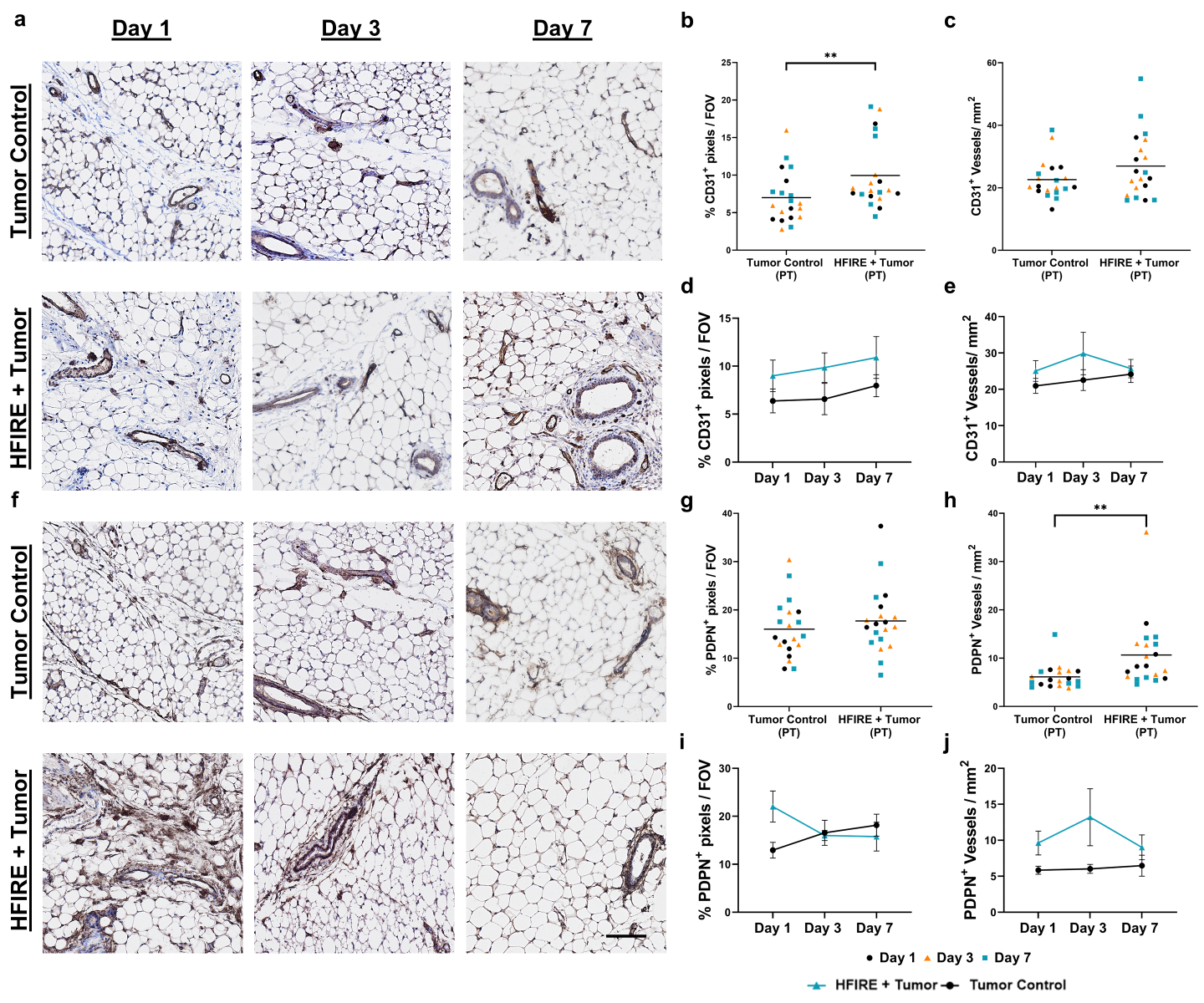


**Supplemental Figure 4. Peritumoral mammary fat pad undergoes microvascular remodeling following SA-H-FIRE.** Representative images from **(a)** CD31 and **(f)** PDPN staining of the peritumoral mammary fat pad separated by day, and treatment respectively. Scale bar represents 50 µm. Area percent coverage of **(b)** CD31^+^ pixels or **(g)** PDPN^+^ pixels per field of view. Cross sectional vessel density of **(c)** CD31 and **(h)** PDPN positive vessels in peritumoral mammary fat pad region. Area percent coverage of **(d)** CD31^+^ and **(i)** PDPN^+^ pixels per field of view separated by day. Vessel density of **(e)** CD31^+^ and **(j)** PDPN^+^ vessels in peritumoral mammary fat pad region separated by day. Data shown Mean ± SEM for separation by day or grand mean for grouped. n = 6-7 animals per group, 40 in total. **p*<0.05, ** *p*<0.01, from two-way ANOVA, with multiple comparison or unpaired T-test.

**Supplementary Figure 5. Collagen remodeling in the viable tumor region. (a)** Whole mount image of the viable region demarcated by the black line surrounding the necrotic areas from a mouse three days post SA-HFIRE treatment. Scale bar represents 2 mm **(i)** representative image of PSR staining in viable tumor region. Scale bar represents 50 µm Area percent coverage of brightfield picrosirius red stain (PSR) in the viable tumor region, **(c)** combined and **(c)** separated by days. **(d)** Ratio of Type I (red-orange) and Type III (green) collagen fibers from polarized light PSR images of the viable tumor region for each day. All data shown as Mean ± SEM. * *p*< 0.05. n = 6-7 animals per group.


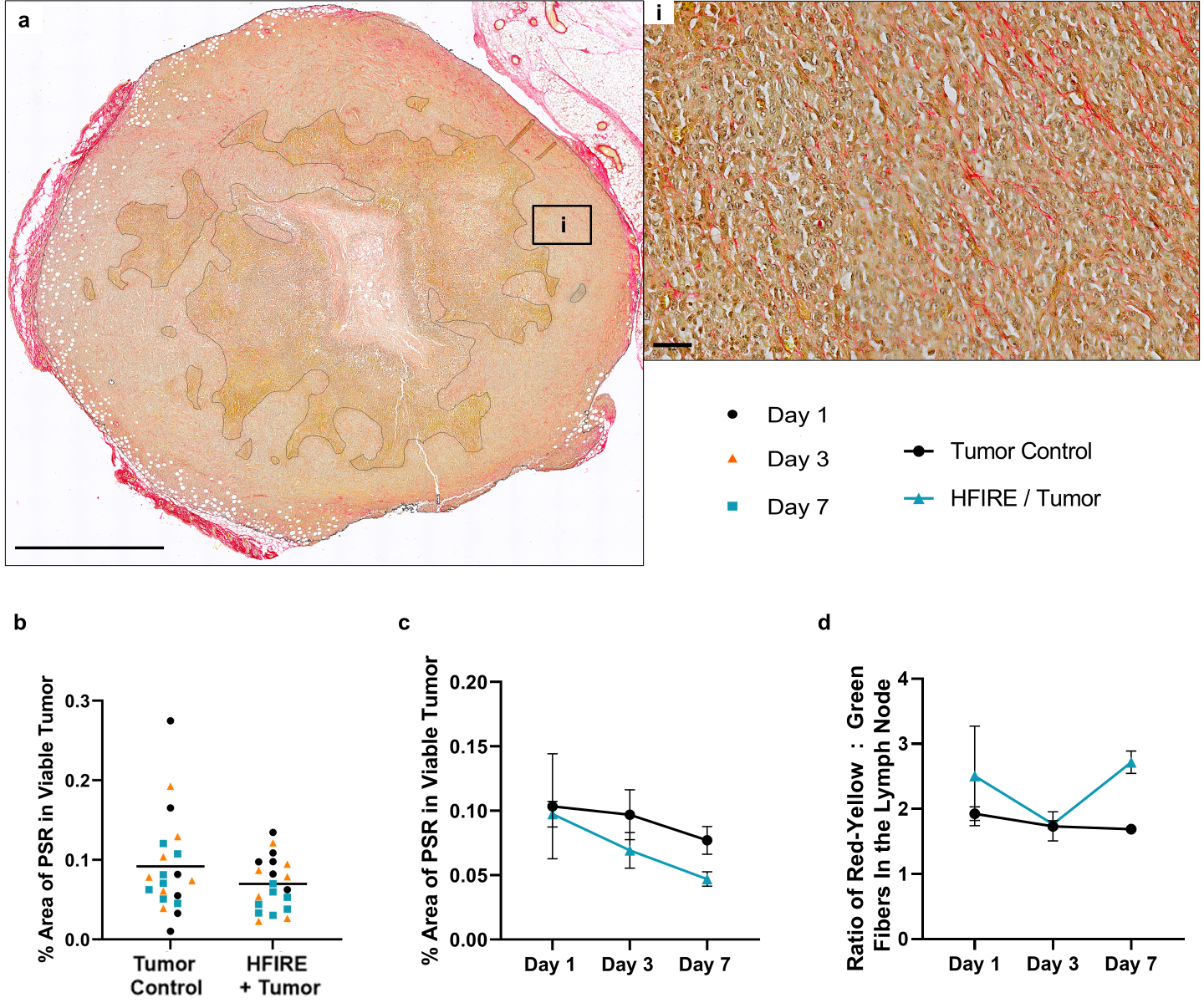


**Supplemental Figure 6. Qiagen Mouse Angiogenesis panel shows increase in CXCL2 gene expression one day post SA-HFIRE treatment**. **(a)** Upregulated and downregulated gene expression in the tumor of SA-HFIRE against tumor control groups using ΔΔCT method. Genes in red are upregulated, while green is downregulated. Genes in black state no difference. Genes with greater than 2-fold change were labeled. **(b)** Fold regulation of *p<0.05* significant genes. Dotted lines represent 2-fold change. n = 6 animals per group.


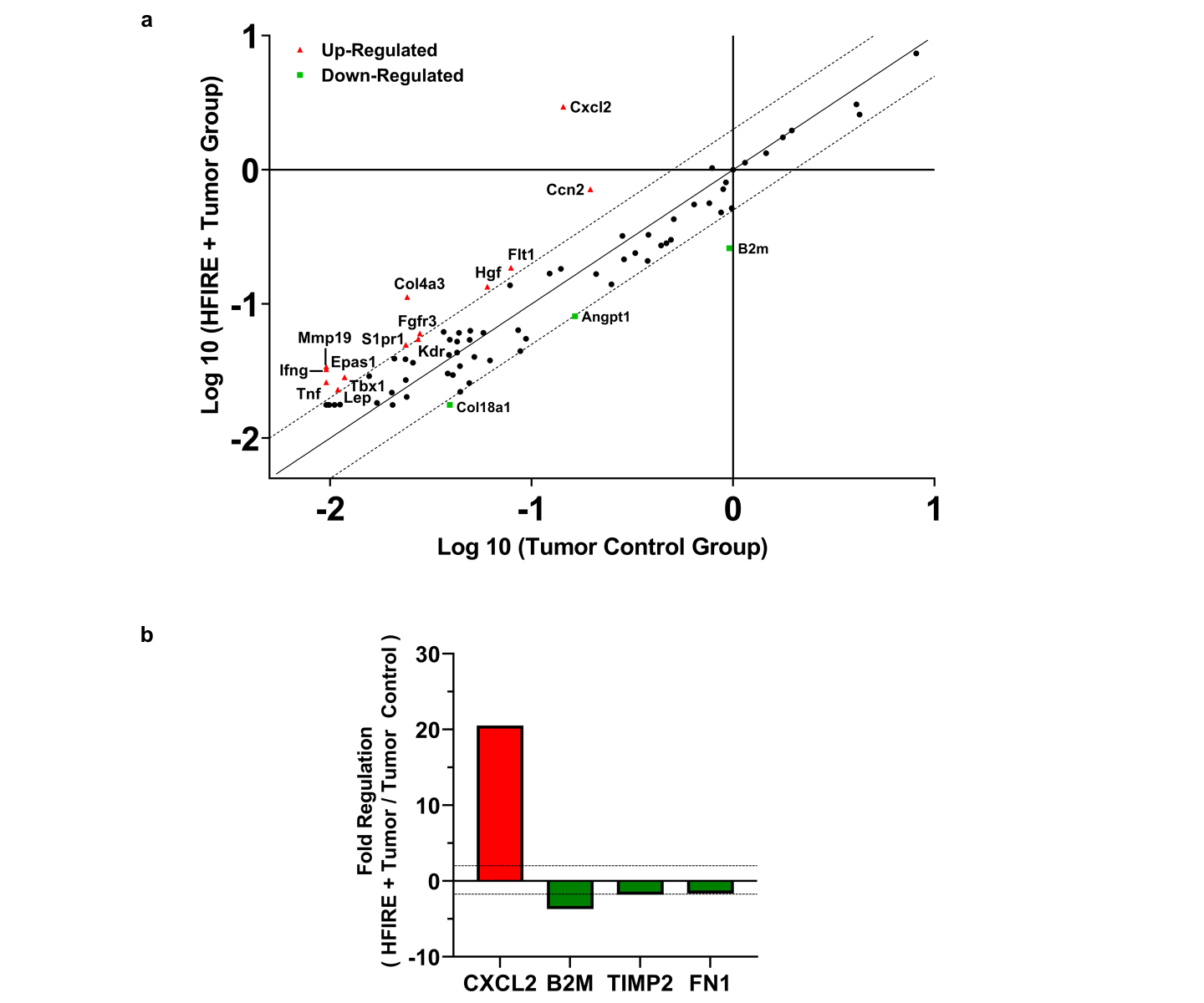


**iSupplemental Figure 7. Gene expression of the mammary fat pad following SA-HFIRE treatment.** Gene expression data in the mammary fat pad as a combination of all the days, for **(a)** VEGFA, **(b)** VEGFC, and **(c)** CCL21. Gene expression data in the mammary fat pad separated by day, for **(d)** VEGFA, **(e)** VEGFC, and **(f)** CCL21. All data is relative to R18S measured using RT-qPCR from Tumor Control and HFIRE + Tumor animals respectively. All data shown as combined or separated by day with Mean ± SEM. * *p*< 0.05 as determined by 2-way ANOVA followed by Tukey's multiple comparisons. n = 3-7 animals per group.


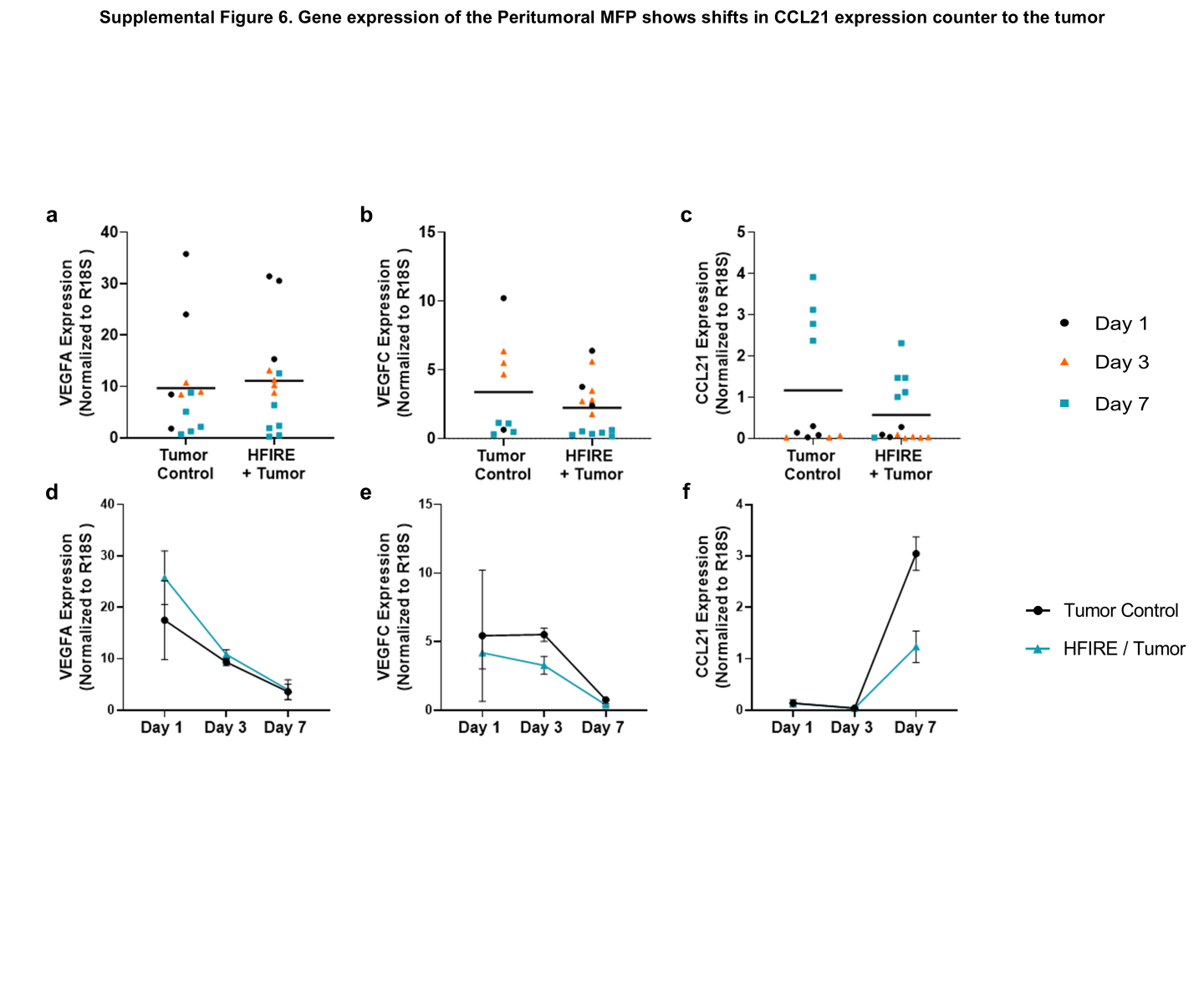


**Supplementary Figure 8. Collagen remodeling in the tumor draining inguinal lymph node. (a)** Inguinal lymph node from a one-day post SA-H-FIRE treatment mouse stained with PSR in brightfield. Scale bar represents 250 µm. **(i)** Representative image of the medullar region where most of the PSR signal is located outside of the capsule. Scale bar represents 50 µm. Area percent coverage of brightfield picrosirius red stain (PSR) in the tumor draining inguinal lymph node, **(b)** combined and **(c)** separated by days. **(d)** Ratio of Type I (red-orange) and Type III (green) collagen fibers from polarized light PSR images of the tumor draining inguinal lymph nodes for each day. All data shown as Mean ± SEM. n = 6-7 animals per group.


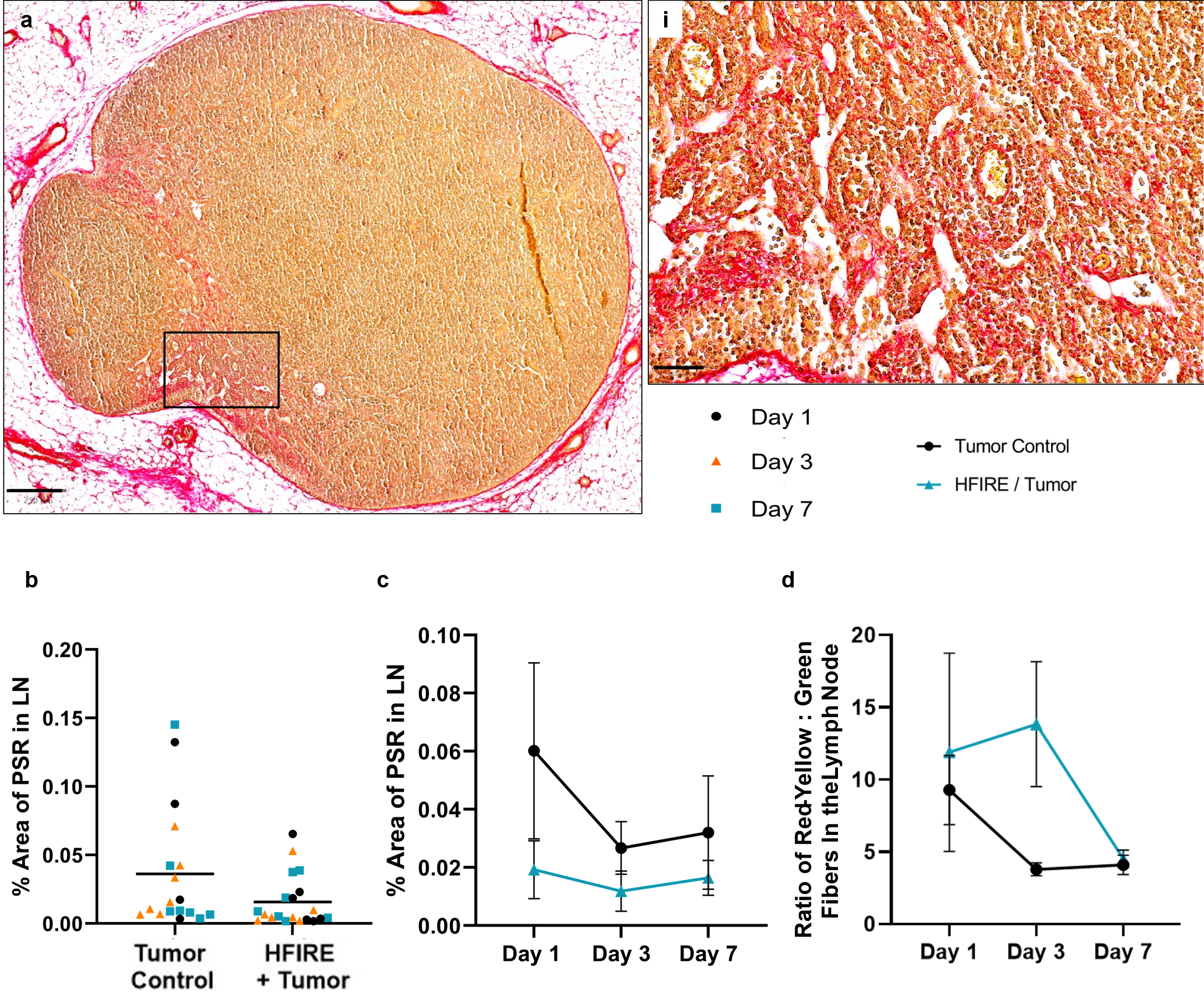


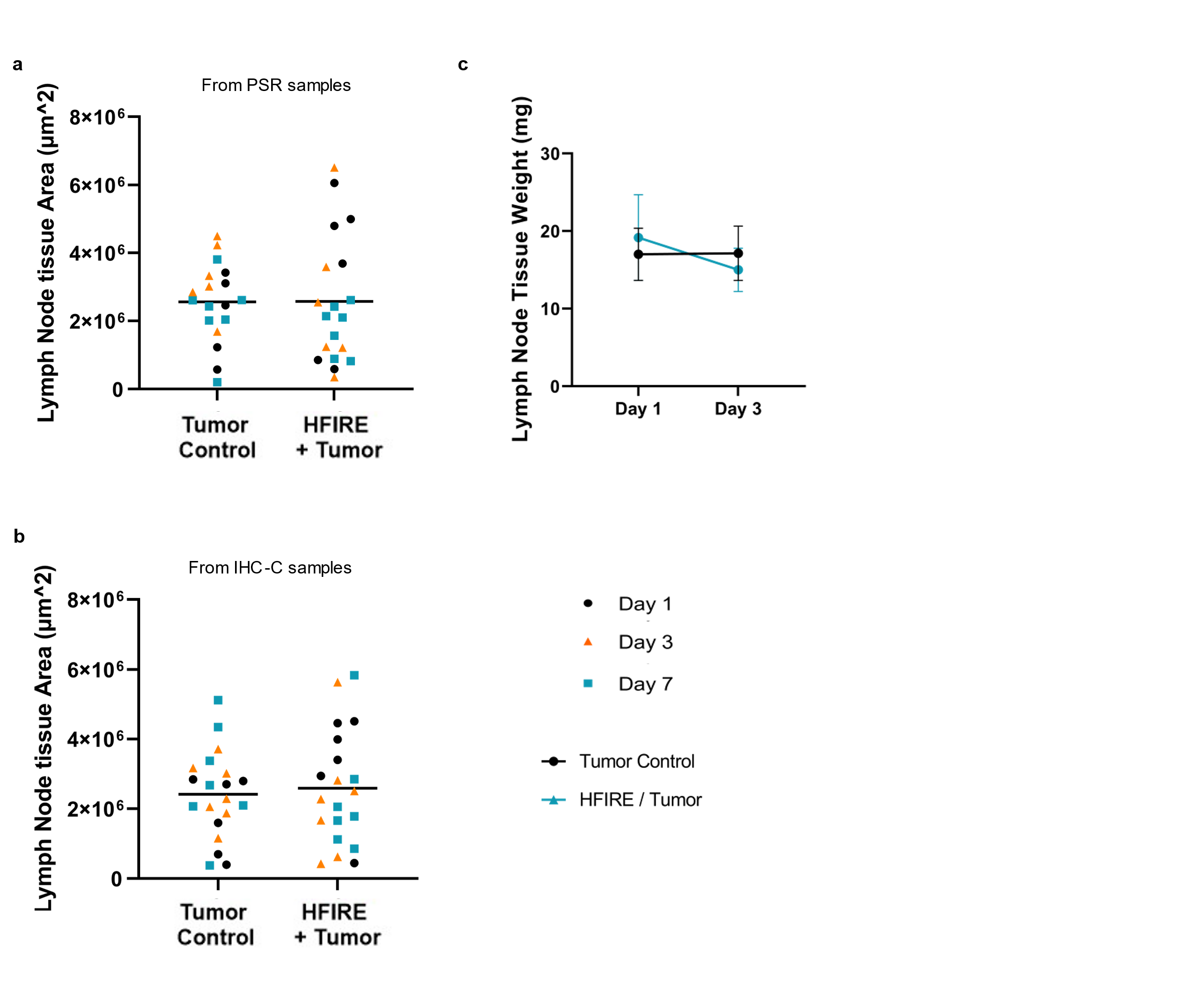


**Supplemental Figure 9.** **Tumor draining inguinal lymph node shows no differences in the cross-sectional areas for treatment groups.** Lymph node cross sectional tissue area used in analysis from **(a)** picrosirius red stained samples and **(b)** Immunohistochemistry-stained samples. **(c)** Axillary lymph node weight used for ELISA analysis. n = 6-7 animals per group.

**Supplemental Table 1: Predicted tumor coverages from COMSOL model.** For all studies : Electrodes with 4 mm exposure, 4 mm spacing, and a 6.5 mm x 5 mm tumor (141.99 mm^3^).

| Voltage Applied (V) | Scheme (µs) | Percentage Volume Coverage |
| --- | --- | --- |
| 600 | 2-5-2-5 | 23.32 % |
| 800 | 2-5-2-5 | 37.49 % |
| 1000 | 2-5-2-5 | 48.83 % |
