# Supplementary material for: Transient Lymphatic Remodeling Follows Sub-Ablative High-Frequency Irreversible Electroporation Therapy in a 4T1 Murine Model
Source: Ann Biomed Eng. 2025 Feb 25;53(5):1148–64. doi: 10.1007/s10439-024-03674-y (PMC12006248; doi:10.1007/s10439-024-03674-y)
Supplement: Supplementary file 2 — Supplementary file2 (DOCX 95 KB) [file 10439_2024_3674_MOESM2_ESM.docx]

**Supplemental Method : H-FIRE Treatment Modeling in COMSOL**

A 3D, time-dependent electric currents model (COMSOL^TM^ Multiphysics 6.1) was created to replicate the electric field distribution through the tumor-bearing fat pad and surrounding tissues. The system was modeled with 3 distinct layers: muscle, fat pad, and skin (Supplemental Fig. 1c), with thicknesses of 3, 7, and 0.25 mm, respectively. The domain widths were 2 cm. The implanted tumor was modeled as a sphere with a 6.5 mm diameter. Values for tissue dimensions are estimated based on average values measured by caliper at the day of treatment. Two insulated sharp-tip needle electrodes were modeled as cylinders with a 0.25 mm radius, a 4 mm exposure, and a 4 mm center-to-center separation. The electric potential, $\Phi$, at the end of an electric pulse was calculated with a modified Laplace equation under the electro-quasistatic approximation:

|  | $-\nabla\cdot\left( \sigma\cdot\nabla\Phi\right)=0$ | (1) |
| --- | --- | --- |

where $\sigma$is the material electrical conductivity. The conductivities for wet skin and the fat pad are 0.15 (S/m) and 0.2 (S/m), respectively[1]. We modeled the muscle as an anisotropic tissue with parallel and perpendicular conductivities of 0.44 (S/m) and 0.12 (S/m), respectively[1]. The conductivity of the electroporated tumor was estimated using methods previously validated[2] and by sweeping through electrical conductivities in COMSOL until the simulated current matched the measured current at the beginning of treatment. We observed that the electric field distribution was not greatly affected by small changes in the chosen tumor conductivity, so we chose the best fit for our ablation volume estimations.

The boundaries of both electrodes in contact with the tissue were set to $\Phi=600 V$ and $\Phi=0$, for the source and sink, respectively. The outer boundary of the tissue was treated as electrically isolated. The resulting electric field was calculated as:

|  | $\vec{E}=-\nabla\Phi$ | (2) |
| --- | --- | --- |

To calculate the percent tumor ablation, we integrated the volume above the 4T1 lethal threshold (1071 V/cm) within the volume of the simulated tumor.

# References

1. Hasgall PA, Di Gennaro F, Baumgartner C, Neufeld E, Lloyd B, Gosselin MC, Payne D, Klingenböck A, and Kuster N. IT’IS Database for thermal and electromagnetic parameters of biological tissues, Version 4.1. , 2022.doi:10.13099/VIP21000-04-1. itis.swiss/database

2. Jacobs, E. J., K. N. Aycock, P. P. Santos, J. L. Tuohy, and R. V. Davalos. Rapid estimation of electroporation-dependent tissue properties in canine lung tumors using a deep neural network. *Biosens Bioelectron* 244:, 2024.
